# Exploring the failing right ventricle in pulmonary hypertension by cardiac magnetic resonance: An in vivo study utilizing Macitentan

**DOI:** 10.1002/pul2.12124

**Published:** 2022-07-01

**Authors:** Gerard Murphy, Geeshath Jayasekera, James Mullin, Lindsay Gallagher, David J. Welsh

**Affiliations:** ^1^ Scottish Pulmonary Vascular Unit Glasgow Caledonian University Glasgow UK; ^2^ Institute of Neuroscience & Psychology University of Glasgow Glasgow UK

**Keywords:** cardiac magnetic resonance imaging, Macitentan, pulmonary arterial hypertension, right ventricle function and dysfunction

## Abstract

Cardiac magnetic resonance (CMR) imaging is used to assess the right ventricle (RV) of pulmonary hypertensive (PH) patients and more recently to track changes in response to therapy. We wished to investigate if repeat CMRs could be used to assess ventricular changes in the Sugen 5416 hypoxic (Su/Hx) rat model of PH treated with the dual endothelin receptor antagonist Macitentan. Male Sprague Dawley Su/Hx rats were dosed for 3 weeks with either vehicle or Macitentan (30 mg/kg) daily, control rats received only vehicle. All rats underwent three CMR scans; before treatment, 2 weeks into treatment, and end of the study. A separate group of Su/Hx and control rats, treated as above, underwent terminal hemodynamic measurements. Using terminal and CMR measurements, Macitentan was found to lower RV systolic pressure pulmonary artery remodeling and increase RV ejection fraction but not change RV hypertrophy (RVH). Repeat CMRs determined that Su/Hx rats treated with Macitentan had significantly reversed RVH via reducing RV mass as well as reducing elevated left ventricular eccentricity index; reductions in RV mass were also observed in Su/Hx vehicle rats exposed to normoxic conditions. We have demonstrated that repeat CMRs can be used to assess the volume and structural changes in the ventricles of the Su/Hx rat model. Using repeat CMRs has allowed us to build a more complete picture of the response of the RV and the left ventricle to treatment. It is unknown if these effects are a consequence of direct action on the RV or secondary to improvements in the lung vasculature.

## INTRODUCTION

Pulmonary arterial hypertension (PAH) is a fatal disease of the pulmonary vasculature characterized by the remodeling of small pulmonary arteries. This involves increased smooth muscle, fibroblast, and endothelial cell proliferation, the latter of which can lead to the formation of plexiform lesions.^1^ This results in increased pulmonary artery pressure, pulmonary vascular resistance, and an increased afterload to the right ventricle (RV).^2^, ^3^ To compensate, the RV increases both its contractility and wall thickness, which is a key indicator of patient survival in PAH.^4^ However, the RV can deteriorate from adaptive to maladaptive remodeling characterized by reduced stroke volume, decreased cardiac output, bowing of the interventricular septum (IVS), and diastolic dysfunction, eventually leading to RV failure.^5^

RV systolic pressure (RVSP) or mean pulmonary artery pressure (mPAP) are commonly obtained in both clinic and animal model studies by invasive right heart catheterization (RHC). A noninvasive method such as echocardiography has been used in the clinic to assess the probability of pulmonary hypertension (PH),^6^ as well as track the progression of disease in monocrotaline models of PH.^7^, ^8^ Cardiac magnetic resonance (CMR) imaging is widely regarded as the gold standard for determining cardiac structure and function.^9^ Determining accurate RV and left ventricle (LV) variables is an advantage that CMR has over other methods of analysis,^10^ notably echocardiography and RHC. The value of CMR in monitoring the effectiveness of drug therapy in human PAH is well established.^9^ PAH patients ultimately succumb to RV failure. Most animal studies looking at preventing or reversing PH with treatment primarily use the Fulton index^11^ to assess end‐stage RV hypertrophy as an outcome measurement. This is a gross measurement that results often in nonsignificant findings due to a wide spread of variables. The use of CMR in animal models could provide more accurate measurements of ventricular volumes and function.^12^ This has been demonstrated recently by our own group in the Su/Hx rat model.^13^

Macitentan is a well‐established dual endothelin receptor antagonist, which helps mitigate the vasoconstrictive and mitogenic effects of elevated endothelin‐1 (ET‐1) in PAH.^14^, ^15^ Macitentan has been shown to reduce or prevent increases in pulmonary vascular remodeling, RV systolic pressure, and RV hypertrophy, assessed by gross histology, in preclinical models of PH, such as monocrotaline^8^, ^16^ and Sugen 5416 (Su) combined with chronic hypoxia (Su/Hx) models of PH,^11^ as well as reducing morbidity and mortality in PAH patients.^15^ Specific human RV changes in response to Macitentan are currently being investigated (REPAIR study—clinical trial ID: NCT02310672).

The present study sought to build on the feasibility of CMR in the Su/Hx rat model by utilizing repeat scans, assessing each rat before treatment and during disease‐targeted therapy using Macitentan and examining the same variables that we use to establish disease severity in humans. To do this, we utilized the established effectiveness of Macitentan as a treatment model in a reversal study of established pulmonary hypertension.

## METHODS

### In vivo study design

See Supporting Information for housing details, Su/Hx rat model/drug treatment, and study outline (Supporting Information: Figure S1). Briefly, 4‐week‐old Sprague Dawley rats (*n* = 38) purchased from Envigo, were initially divided into two groups. An Su/Hx group (*n* = 24) and a control group (*n* = 14). The Su/Hx group was administered a subcutaneous injection of Su (20 mg/kg). Rats were then placed for 3 weeks in hypobaric hypoxic conditions (550 mbar), followed by 2 weeks of normoxic conditions (1013 mbar) (Nx) to allow for PH development. Control (normoxic) group rats initially received a subcutaneous injection of the Su vehicle, then were kept in Nx conditions for 5 weeks. To demonstrate the development of PH, both control and Su/Hx rats (*n* = 4 for each group) were assessed hemodynamically 5 weeks into the protocol. The remaining Su/Hx rats (*n* = 20) subsequently received either Macitentan (30 mg/kg) or its vehicle (7.5% gelatin) via daily oral gavage for a further 3 weeks, control rats (*n* = 10) only received vehicle. For the CMR arm of the study, the rats were divided into Su/Hx with vehicle (*n* = 6), Su/Hx with Macitentan (*n* = 6), and control with vehicle (*n* = 6). For the hemodynamic arm, the rats were divided into the same groups (*n* = 4 per group).

### CMR protocol and analysis

CMR measurements were taken at 5 weeks (baseline) after the establishment of a PH phenotype. Repeat CMRs were then taken, on the same rats, at 2 and 3 weeks post commencement of Macitentan or vehicle treatment. CMR was carried out with a Bruker Pharmascan 7T system (Bruker). Rats were anesthetized with isoflurane for induction at a rate of 3% (v/v) in a 5% (v/v) mix of 1:1 O_2_ and N_2_O; for maintenance, the isoflurane was lowered from 2% to 2.5% (v/v). During the procedure, body temperature, electrocardiography, and breathing rate were measured. Gating for CMR was achieved with electrocardiography electrodes. An external water jacket was used to maintain a core temperature. After preliminary long and short axis scans to confirm the correct positioning of the rat, long axis cine scans (four chambers) were carried out to set the angle of the subsequent short axis cine scan (two chambers). The images were acquired using a slice thickness of 1.5 mm ensuring that the entirety of both ventricles was covered; examples of long and short axis cine images are available in the (Supporting Information: Figure S2). The CMR parameters were as follows: field of view—30.00 mm × 30.00 mm, image resolution—192 × 192 pixels, flip angle—15°, echo time—3.08 ms, Rep. time—8.36 ms, number of phases—25, number of averages—4 to 6 and software version—Paravision 5.1. Scans were analyzed blinded to rodent treatment/conditions. Manual planimetry using OsiriX (Pixmeo SARL) determined epicardial and endocardial borders at both end‐diastolic and end‐systolic frames leading to the determination of RV and LV, end‐diastolic volume (EDV), and ESV. These led to the calculation of systolic volume (SV) (SV = EDV − ESV), ejection fractions for each ventricle (EF% = SV/EDV × 100%), and cardiac output (CO = SV x heart rate). Trabeculations and papillary muscles were considered part of the blood pool. RV and LV plus septum masses were also examined at end‐diastolic and end‐systolic frames to determine RV hypertrophy (RVH) via the Fulton index (RV/LV + S). LV eccentricity index (LVEI) is a measure of the displacement of the interventricular septum (IVS); it was measured as the ratio of minor axis diameter parallel to the septum and minor axis diameter perpendicular to septum,^17^ taken at both systole and diastole. All volume and mass measurements were indexed to total body surface area (TBSA) (cm^2^). Supporting Information: Tables S1–S3 tabulate CMR data from baseline, and 2‐ and 3‐week scans, respectively. Further analysis of seven randomly selected scans was carried out by a second observer to assess interobserver variability (Supporting Information: Table S4). One rat died under anesthetic when undergoing its final CMR scan.

### Assessment of hemodynamics

Rats were induced with 3%–4% (v/v) isoflurane and maintained at 3% (v/v) isoflurane mixed with medical oxygen (1 L/min). Using the Mikro‐Tip® Pressure Volume Ultra Foundation System (ADInstruments), a pressure‐volume catheter (SPR‐869; Millar instruments) was inserted into the RV via the jugular vein to determine RVSP. Systemic arterial pressure (SAP) pressures were measured by inserting a catheter into the carotid artery. Data gathered was analyzed using LabChart Pro 7 software (ADInstruments). After the procedure, the heart was removed, and RV hypertrophy was determined by comparing the weight of the RV‐free wall to the LV plus the IVS (Fulton index).

### Histology

After final CMRs, rats were euthanized via anesthetic overdose, then the left lung was fixed in 10% neutral buffered formalin for histology. The lungs were then embedded in paraffin wax followed by cutting into 5 µm sections and stained with Millar elastin counterstained with picrosirius red. Pulmonary arteries containing a distinct double elastic lamina visibly around at least half the diameter of the vessel were constituted as a remodeled vessel. Vessels were counted in a blinded fashion over two separate occasions to give an average number of nonremodeled, remodeled, and total vessels. The extent of remodeling was calculated as a percentage of remodeled vessels compared to the total number of vessels. Images were captured using an EVOS XL core microscope (ThermoFisher Scientific).

### Brain natriuretic peptide (BNP) enzyme‐linked immunosorbent assays (ELISAs)

BNP plasma levels were quantified by ELISA (Abcam) according to the manufacturer's instructions.

### Statistical analysis

All data are expressed as mean ± standard error of the mean. All statistical tests were performed using GraphPad Prism 8 (GraphPad) or SPSS statistics 25 (IBM), where stated. Intergroup comparisons were analyzed with a two‐tailed Student's unpaired *t*‐test where two independent groups are being compared. For >2 groups a one‐way analysis of variance (ANOVA) followed by Tukey's post hoc test was carried out. Intragroup data were analyzed using repeated measures one‐way ANOVA/mixed effects analysis (as stated) followed by Tukey's post hoc test. A probability level of *p* < 0.05 was defined as being statistically significant. Interobserver variability of CMR analysis was assessed by intra‐class correlation coefficient using a two‐way mixed effects model with an absolute agreement and average measures using SPSS statistics 25 (Supporting Information: Table S4).

## RESULTS

### Establishment of PH phenotype in Su/Hx rats

A baseline CMR was taken at the 5‐week point of the Su/Hx protocol, this demonstrated that Su/Hx caused a significant decrease in right ventricular ejection fraction (RVEF) in relation to the control group (Supporting Information: Figure S3A). Accompanying this, there was a significant increase in RVH, due to increased RV mass, when compared to control rats (Supporting Information: Figure S3B,C). Separately, hemodynamic and histology assessment at baseline demonstrated increased RVSP, RVH (Supporting Information: Figure S4A,B), and pulmonary vascular remodeling (Supporting Information: Figure S4D) mediated by Su/Hx.

### Hemodynamic and histological assessment of Su/Hx rats treated with Macitentan

**Figure 1:**
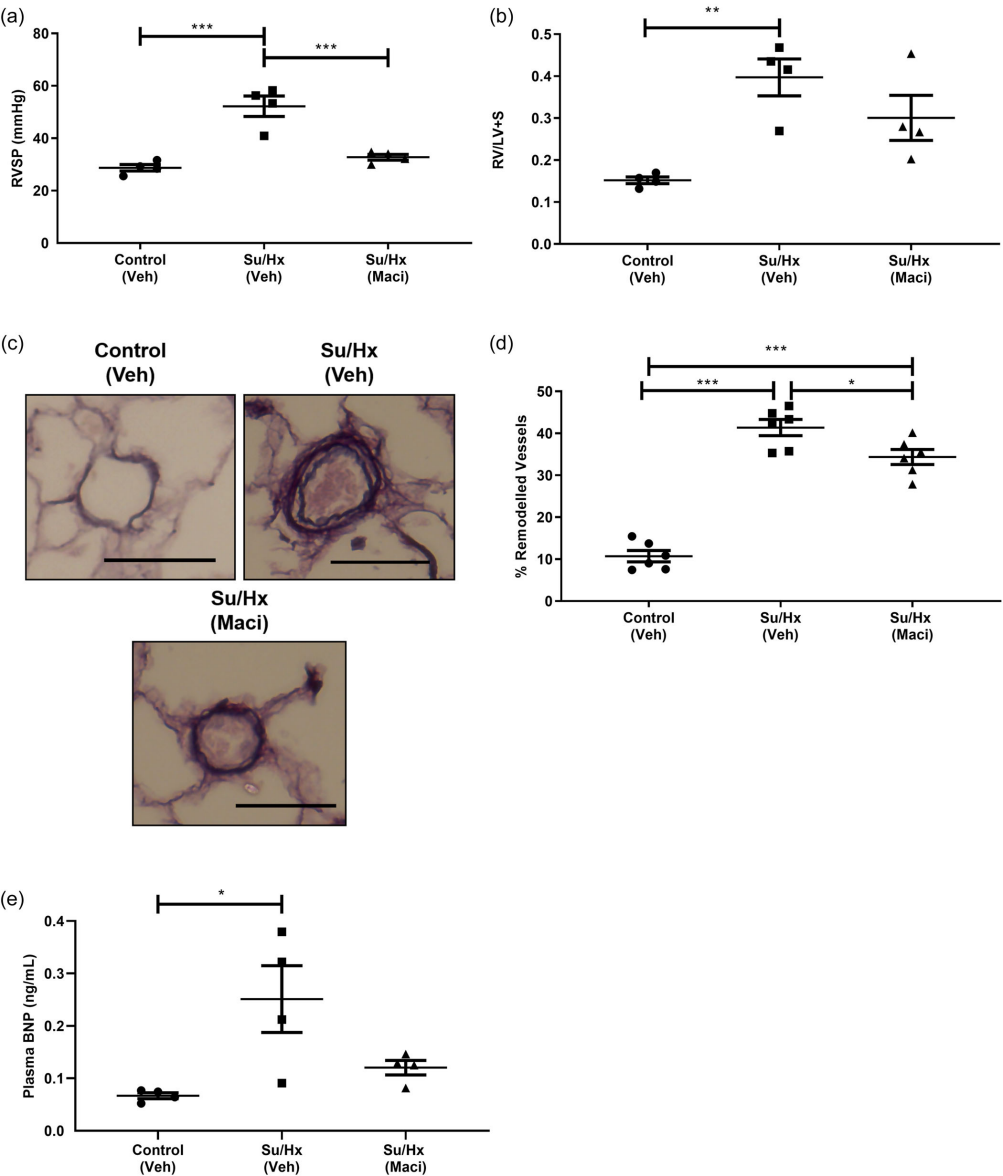
Effect of 3‐week Maci or Veh treatment on Su/Hx‐induced PAH determined by terminal hemodynamic measurements and postmortem histology of CMR rats. (a) RVSP, (b) RV hypertrophy, (c) representative micrograph of Control Veh, Su/Hx Veh, and Su/Hx Maci, (d) % remodeled vessels, and (e) BNP plasma levels assessed by ELISA. Data represented as mean ± standard error of the mean. *n* = 4 for RVSP, RV hypertrophy, and BNP ELISA, *n* = 6 for % remodeled vessels, *p < 0.05, **p < 0.01, ***p < 0.001 as indicated, determined by one‐way analysis of variance with Tukey's post hoc analysis, scale bar in each micrograph = 50 µm. BNP, brain natriuretic peptide; CMR, cardiac magnetic resonance; ELISA, enzyme‐linked immunosorbent assay; Maci, Macitentan; PAH, pulmonary arterial hypertension; RV, right ventricle; RVSP, RV systolic pressure; Veh, vehicle.

Su/Hx rats developed increased RVSP and RVH compared to control rats. RVSP was reversed by 3‐week Macitentan therapy; however, postmortem RVH was not (Figure 1a,b). Treatment conditions had no effect on either SAP or heart rate (Supporting Information: Figure S5). Histology carried out after final CMRs found Macitentan had partially reversed Su/Hx‐mediated increase in pulmonary vascular remodeling (Figure 1c,d). BNP levels were found to be significantly elevated in Su/Hx rats versus control rats. Macitentan treatment did not significantly reverse this increase (Figure 1e).

### Effect of Macitentan on Su/Hx right ventricle function and structure using CMR

**Figure 2:**
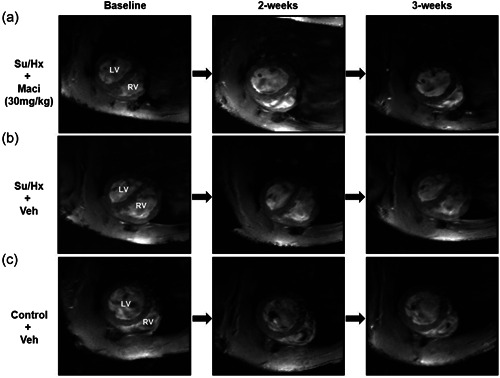
Representative CMR short axis cine images taken at diastole of baseline, and 2 and 3‐week CMRs treated with either Maci or Veh. Su/Hx rats with Macitentan (a), Su/Hx vehicle, (b) and Control vehicle (c). LV and RV identified. Short axis cine images were acquired using a slice thickness of 1.5mm ensuring that the entirety of the biventricular length was covered. CMR, cardiac magnetic resonance; LV, left ventricle; Maci, Macitentan; RV, right ventricle; Veh, vehicle.

**Figure 3:**
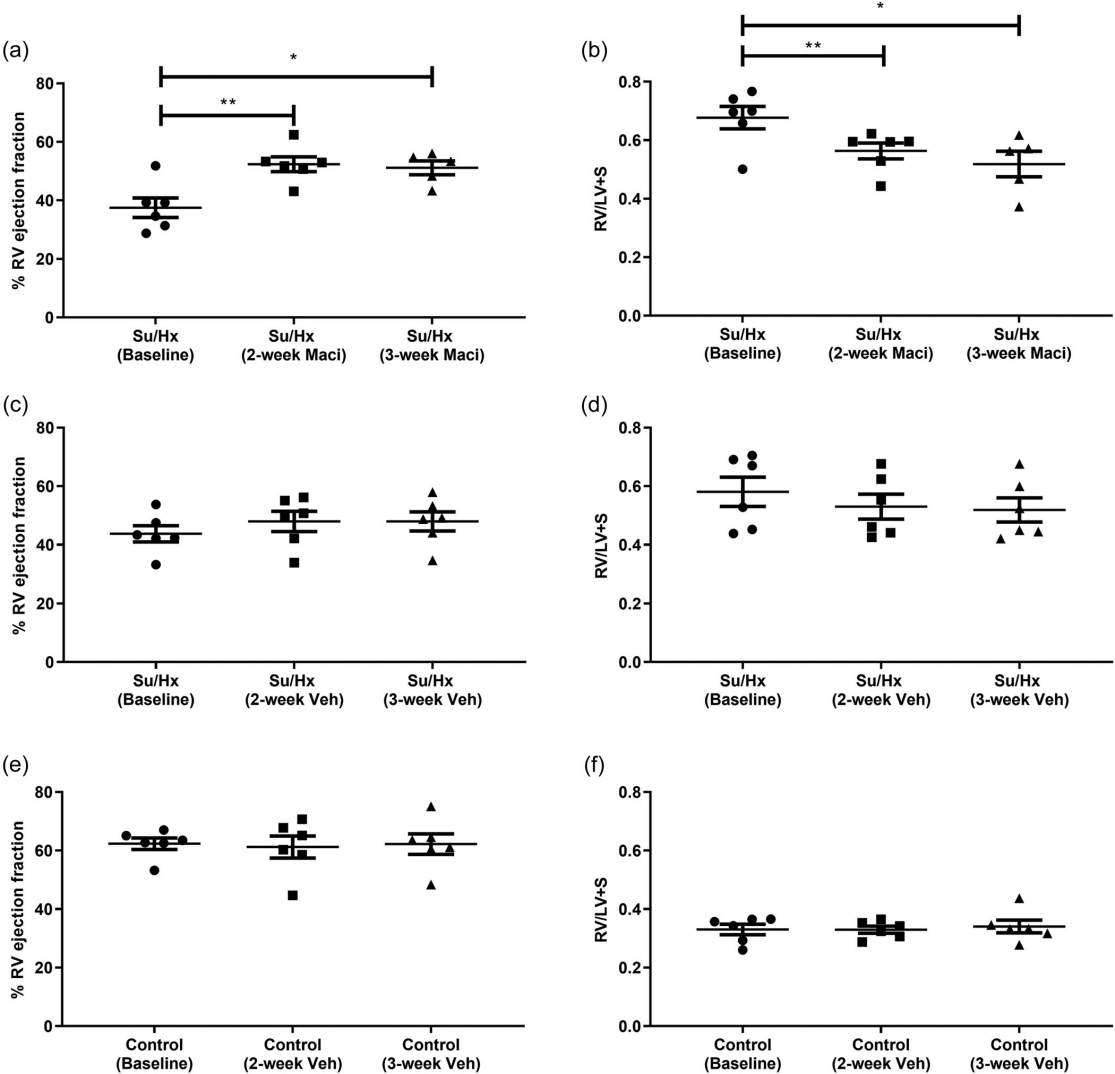
Repeat CMR analysis of the effect of Maci or Veh on RVEF and RV hypertrophy (RV/LV+ S) across 3‐week treatment. Su/Hx with Macitentan (a) RVEF and (b) RV hypertrophy. Su/Hx vehicle (c) RVEF and (d) RV hypertrophy. Control vehicle (e) RVEF and (f) RV hypertrophy. Data represented as mean ± standard error of the mean. *n* = 5–6, *p < 0.05, **p < 0.01, as indicated, determined by repeated measures one‐way analysis of variance or mixed effects model with Tukey's post hoc analysis. CMR, cardiac magnetic resonance; LV, left ventricle; Maci, Macitentan; RV, right ventricle; RVEF, RV ejection fraction; Veh, vehicle.

**Figure 4:**
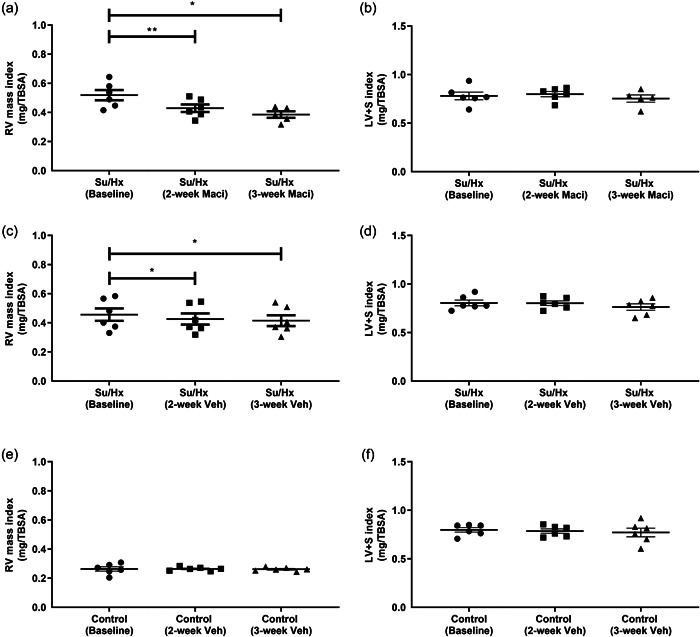
Repeat CMR analysis of the effect of Maci or Veh on RV and LV plus septum mass indexed to TBSA (cm^2^). Su/Hx with Maci (a) RV mass index and (b) LV + S mass index. Su/Hx vehicle (c) RV mass and (d) Su/Hx vehicle LV + S mass index. Control vehicle (e) RV mass index and (f) LV + S mass index. Data represented as mean ± standard error of the mean. *n* = 5–6, *p < 0.05, **p < 0.001 as indicated, determined by repeated measures one‐way analysis of variance or mixed effects model with Tukey's post hoc analysis. CMR, cardiac magnetic resonance; LV, left ventricle; Maci, Macitentan; RV, right ventricle; RVEF, RV ejection fraction; Veh, vehicle; TBSA, total body surface area.

**Figure 5:**
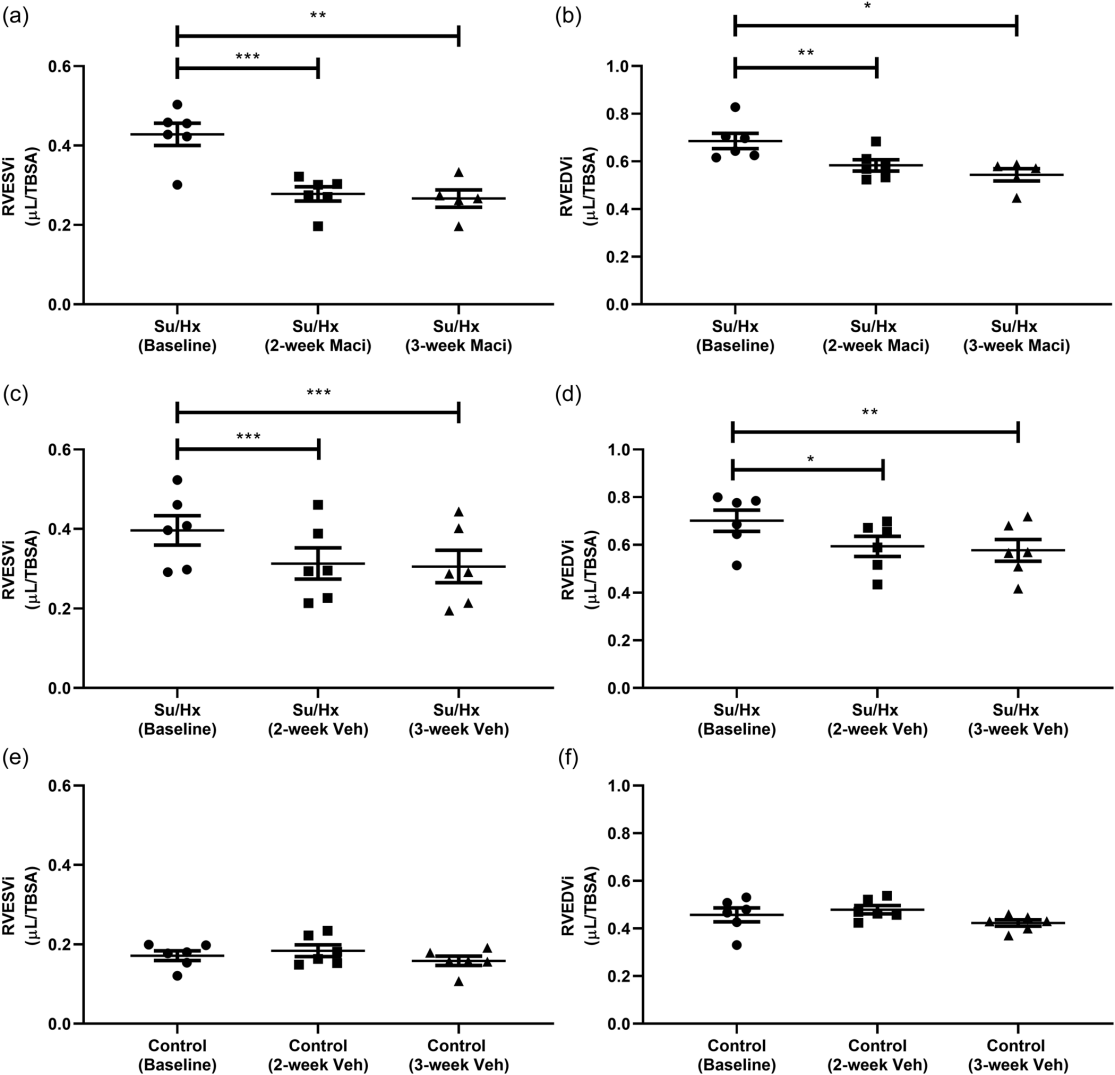
Repeat CMR analysis of the effect of Maci or Veh on RVESVi and RVEDVi across 3‐week treatment, volumes are indexed to TBSA (cm^2^). Su/Hx with Maci (a) RVESVi and (b) RVEDVi. Su/Hx vehicle (c) RVESVi and (d) RVEDVi. Control vehicle (e) RVESVi and (f) RVEDVi. Data represented as mean± standard error of the mean. *n* = 5–6, *p < 0.05, **p < 0.01, ***p < 0.001 as indicated, determined by repeated measures one‐way analysis of variance or mixed effects model with Tukey's post hoc analysis. CMR, cardiac magnetic resonance; Maci, Macitentan; RVEDVi, right ventricular end‐diastolic volume index; RVESVi, right ventricular end‐systolic volume index; Veh, vehicle; TBSA, total body surface area.

When using the CMRs for intergroup comparisons we found that only Su/Hx rats had significantly decreased RVEF compared to control rats, at the 3‐week CMR (Supporting Information: Figure S6A). Both RVH and RV mass index were unchanged with Macitentan treatment when compared between the treatment groups (Supporting Information: Figure S6B,C). By using repeat CMRs, we could determine intragroup changes. Representative short‐axis cine images at diastole highlight the changes from baseline through to the final 3‐week CMR observed in Su/Hx with Macitentan, Su/Hx, and control rats, respectively (Figure 2). These changes are also observed in the representative cine videos of baseline, and 2‐ and 3‐week CMRs across the different treatment groups (Supporting Information: Video [Link], [Link], [Link]). We observed that Macitentan treatment improved RVEF progressively from the baseline scan through to 2‐ and 3‐week treatment (Figure 3a), complemented by significant reductions in RVH (Figure 3b). There were no significant changes in the control and Su/Hx rat's RVEF compared to baseline (Figure 3c–f). RVH reduction was then affirmed as an RV mass index‐specific reduction in Macitentan; however, we also observed a significant reduction in Su/Hx vehicle rats (Figure 4a–d). Both right ventricular end‐systolic volume index (RVESVi) and right ventricular end‐diastolic volume index (RVEDVi) were reduced in Macitentan‐treated Su/Hx rats at 2 and 3 weeks (Figure 5a,b). Interestingly, RVESVi and RVEDVi were significantly reduced in Su/Hx rats at 2 and 3 weeks from baseline (Figure 5c,d). Both RVESVi and RVEDVi did not change in control rats (Figure 5e,f). Both RV stroke volume index and cardiac index (RVCi) were unchanged in control and Su/Hx vehicle rats; however, we did observe a small significant increase in RVCi in Macitentan rats between baseline and 2‐week CMR (Supporting Information: Figure S7).

### Su/Hx and Macitentan: Impact on left ventricle function using CMR

No significant changes in LV ejection fraction were observed in Su/Hx Macitentan‐treated or control rats (Supporting Information: Figure S8A–C). Macitentan treatment did not significantly change either left ventricular end‐systolic volume index (LVESVi) or end‐diastolic volume index (LVEDVi) (Supporting Information: Figure S9A,B), although LVEDVi was found to significantly increase after 3 weeks in Su/Hx with vehicle group compared to 2 weeks (Supporting Information: Figure S9D). Also, LVEDVi was reduced in control rats between 2 and 3 weeks compared to the baseline scan (Supporting Information: Figure S9F). No changes in left ventricular stroke index or cardiac index were observed in any Su/Hx or control rats (Supporting Information: Figure S10A–E).

**Figure 6:**
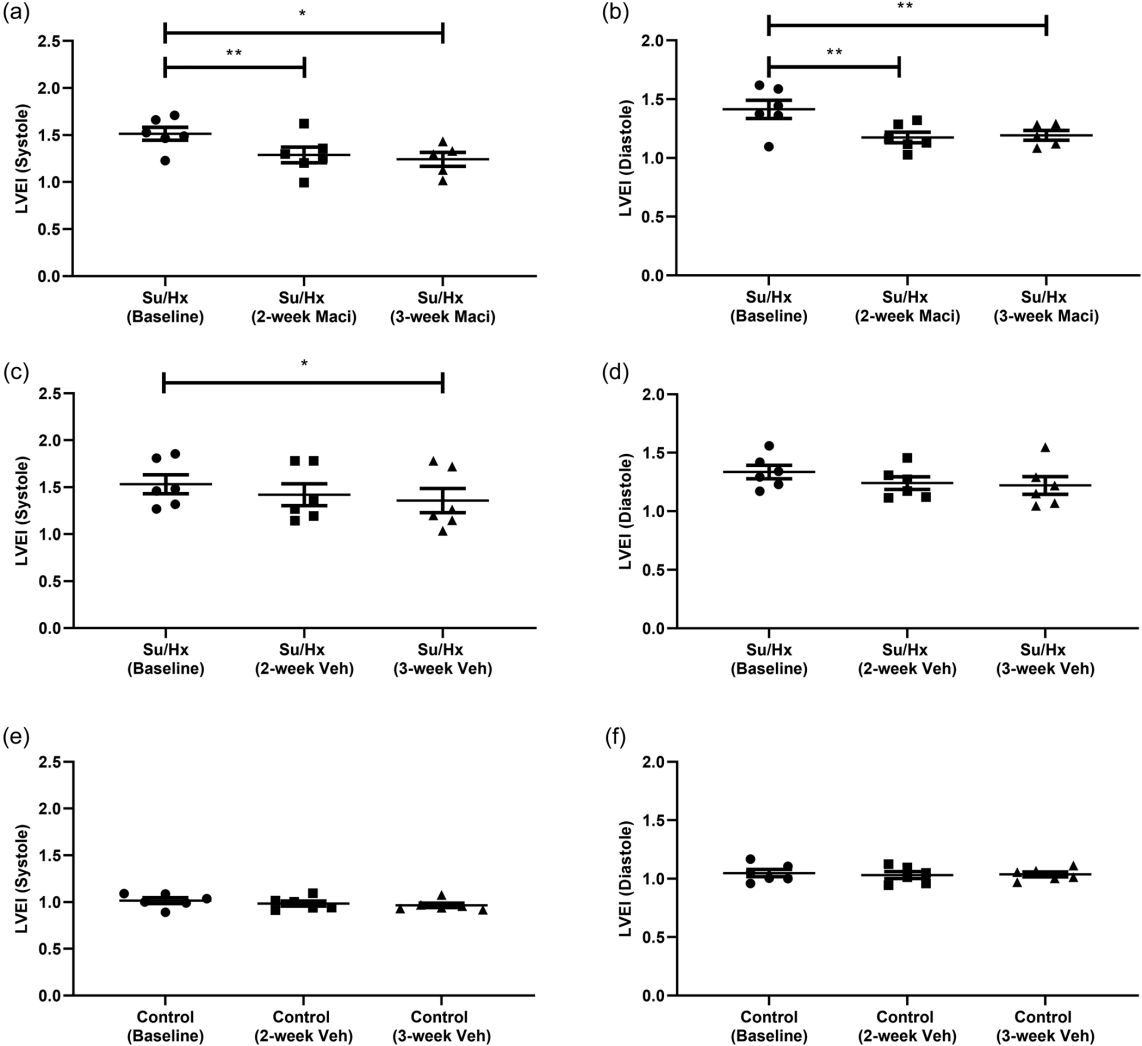
Repeat CMR analysis of the effect of Maci or Veh on LVEI. Su/Hx with Macitentan (a) systole LVEI and (b) diastole LVEI. Su/Hx vehicle (c) systole LVEI and (d) diastole LVEI. Control vehicle (e) systole LVEI, (f) diastole LVEI. Data represented as mean ± standard error of the mean. *n* = 5–6, *p < 0.05, **p < 0.01 as indicated, determined by repeated measures one‐way analysis of variance or mixed effects model with Tukey's post hoc analysis. CMR, cardiac magnetic resonance; LVEI, left ventricular eccentricity index; Maci, Macitentan; Veh, vehicle.

LVEI at systole and diastole was found to be significantly elevated at baseline in Su/Hx groups (Supporting Information: Figure S11A,B). Intergroup comparison of CMRs found that Macitentan had no significant effect on LVEI at systole and diastole (Supporting Information: Figure S11C,F). However, at 3‐week CMR, we observed that Su/Hx rats were still significantly different from control rats at systole, whereas Macitentan‐treated rats were not (Supporting Information: Figure S11E). Using repeat CMRs, we can track LVEI within treatment groups. Treatment with Macitentan reversed high baseline LVEIs at systole and diastole at 2 and 3 weeks (Figure 6a,b), respectively. In Su/Hx, there was a significant reduction in LVEI at systole but no significant change at diastole (Figure 6c,d). There was no change in LVEI of control rats (Figure 6e,f).

## DISCUSSION

In this study, we have demonstrated using serial CMR that we can longitudinally assess RV and LV functional and structural changes throughout Macitentan treatment in the Su/Hx model of PH. Using repeat CMRs has allowed us to build a more complete picture of the response of the RV and the LV to treatment with Macitentan replicating clinical studies of pulmonary hypertension treatment.

When determining the effectiveness of treatment on PH, animal studies have primarily relied on RHC and postmortem ventricular weights/histology. They do not take into account the primary cause of morbidity and mortality in PAH patients: RV failure.^18^ Repeat measurements assessing structure and function in the RV have been studied using echocardiography;^19^, ^20^ however, the size and crescent shape of the RV relative to the LV can make accurate echocardiography difficult.^21^ Studies involving CMR have, to date, focused on observing animal model disease severity^22^, ^23^ and/or end‐stage treatment outcomes.^24^ In human PAH, there are several drug trials ongoing, which are looking to determine specific RV changes in response to Macitentan (REPAIR study—clinical trial ID: NCT02310672) and Riociguat (REPLACE study—ID: NCT02891850). To our knowledge, this is the first preclinical paper that combines repeat CMR scans with drug therapy to assess longitudinal RV and LV changes.

This current study followed an established Su/Hx protocol.^25^ After the disease had been established (baseline), the rats were then treated with drugs or vehicles for further 3 weeks. Our Su/Hx rat model replicated the lower RVEF observed in a modified Su/Hx mouse protocol carried^22^ as well as within clinic in PAH patients.^26^ Previously, our group had undertaken CMRs in Su/Hx rats,^13^ which found no changes in RVEF. Possible reasons for this difference are the previous study had a shorter 2‐week hypoxic phase and it is previously been published that increasing the duration of hypoxia significantly modifies PH indices in Su/Hx rats.^27^ Second, the CMR measurements were taken on different rats, which may increase variability and contribute to the lack of RVEF changes. The initial study helped inform our protocol for the current study.

Using terminal right heart catheterization and histology, we observed that Macitentan reduced RVSP to a nonsignificant difference compared to control levels and reduced pulmonary vascular remodeling compared to Su/Hx alone. This is consistent with the effects of Macitentan observed in another Su/Hx^11^, ^28^ and monocrotaline model.^16^ Interestingly, our hemodynamic study demonstrated no significant decrease in postmortem RV hypertrophy when treated with Macitentan. This is both supported^20^ and contradicted^11^ by previously published research. All these studies used the same strain of rats and the same Su/Hx protocol. This variability, despite animal models’ relative homogeneity, highlights the potential shortcomings of single end‐stage measurements due to differing degrees of disease severity within groups and how this may affect treatment outcomes. This demonstrates a need for repeat measurements of disease and treatment progression in models of PH. This would also more accurately reflect the approach to the heterogeneity of the human PH population. This was exemplified in the current study where the use of repeat CMRs on the same rats, demonstrated that Macitentan treatment does reduce Su/Hx elevated RVH, specifically through a reduction in RV mass but this was also observed in Su/Hx vehicle‐treated rats. Changes in both vehicle‐ and Macitentan‐treated groups suggest a partial recovery from the Su/Hx model; this observation would be missed if we only used the CMRs as an end‐stage time point measurement to compare across treatment groups. In PH patients, RVEF is an important marker of ventricular adaptability, where changes correlate with ventricular compensation or failure.^29^ We observed that Su/Hx reduced RVEF, which was then partially recovered by Macitentan treatment. Similarly, repeat echocardiography in Su/Hx rats also demonstrated increased RVEF in response to Macitentan treatment.^20^

Elevated RVESVi and RVEDVi are important prognostic markers in PAH.^30^ Although we observed a significant decrease in RVESVi and RVEDVi in both Su/Hx‐ and Su/Hx Macitentan‐treated groups, the drop in both volumes were greater in Macitentan‐treated rats. Reductions in both RVESVi and RVEDVi in PH patients have been shown previously with different drug combinations.^31^, ^32^ This compliments the reductions we observed with Macitentan therapy; however, it does not explain why there was a reduction in the absence of Macitentan. Returning Su/Hx rats to normoxic conditions was found to lead to a drop in RVSP and an increase in tricuspid annular plane systolic excursion in the absence of drug intervention.^27^ Additionally, after remaining under normoxic conditions for 10 weeks the Su/Hx mouse model demonstrated a partial normalization of RVSP and RV hypertrophy.^33^ These studies suggest that the return to normoxic conditions partially contributes to a normalization of RVESVi and RVEDVi. Two key indicators of human PAH development are reduced stroke volume and cardiac output;^5^, ^29^ however, we observed no significant changes in both indices measured by CMR. Other in vivo studies have also demonstrated Su/Hx resulting in lowered stroke volume or cardiac output.^11^, ^34^ In one of these studies, Macitentan was found to reverse the depressing stroke volume and cardiac output assessed by echocardiography.^11^ However, similar to our study lower stroke volume and cardiac output were not consistently found in the Su/Hx model of PH.^35^ As explained previously, our use of repeat CMR may be emphasizing inherent variability in the Su/Hx model of PH. Interestingly, our study demonstrates signs of both a failing RV with decreased RVEF, RV dilatation, and a compensated RV where increased RVH helps to maintain cardiac output/index, highlighting the Su/Hx models’ similarity to human PAH but it cannot fully recapitulate the disease.

PAH development leads to RV maladaptation and failure, therefore the research focused on the RV; however, it cannot be looked at in isolation. It is important to consider that both the RV and LV are interdependent, this is in part due to the IVS.^36^ Leftward bowing of the IVS in PAH patients has been previously described,^37^ we observed this at both systole and diastole in Su/Hx rats, measured by the LVEI.^17^ In patients, IVS displacement was identified as an indicator of PAH severity,^38^, ^39^ with LVEI at diastole was identified as a predictor of poorer patient outcome.^40^ Within this study, as with RVH and RV mass using CMRs as a single endpoint measurement would lead to a conclusion that Macitentan does not significantly reverse Su/Hx mediated increases in LVEI. By using repeat CMRs we observed that Macitentan reversed the displacement of the IVS at both systole and diastole suggesting a lowering of RV pressure, which is supported by our terminal hemodynamic data. Clinically, combination therapy with ERA and PDE‐5 inhibitors was effective at reversing increases in diastolic LVEI.^41^ We also observed a partial reduction in LVEI in Su/Hx vehicle rats, which when combined volume and mass data do suggest a partial recovery in PH indices in absence of therapy.

Several animal models of PH have been identified as capturing the change from adaptive RV remodeling to maladaptive remodeling.^7^, ^42^ In our study, we observed hallmarks of adaptive RV remodeling, increased RV mass, increased end systolic and diastolic volumes, and signs of RV dilatation.^43^, ^44^ To mark the progression to maladaptive RV remodeling potentially leading to a point of non‐recoverable RV remodeling (RV failure), we would expect our serial CMR scans to show sustained reductions in RV stroke index potentially contributing to right ventricular‐pulmonary arterial uncoupling as well as a sustained reduction in RV cardiac index.^44^, ^45^, ^46^ Interestingly, we did observe lower RVEF, which is another sign of a maladaptive/failing RV.^29^, ^47^ This was only significantly reversed by treatment with Macitentan but did not completely recover to levels seen in control rats. In addition to CMR scans, we did not observe any outward clinical signs of RV failure like ascites or congestive hepatopathy.^48^ The lack of these clinical signs highlight the gap between this Su/Hx model of PH and the disease itself.

In conclusion, we have demonstrated that the use of repeat CMR is a feasible method for determining RV and LV variables in the Su/Hx rat model of PH. The high fidelity of variables obtained would have been difficult to achieve by the use of only traditional 2D‐echocardiography or RHC.^9^ We have demonstrated that through repeat CMRs, Macitentan can improve RV structure and function in the Su/Hx rat, this would have been missed with single end‐point measurements.

Limitations of this study are that, as a repeat CMR study, a large quantity of data is produced, and the study may not be adequately powered to accurately analyze every measurement. As this study was a proof of concept of repeat CMR measurements in the animal model of PH, future studies may require a greater n number to solve this issue. Additionally, the Su/Hx model used in this study does seem to partially improve both in trend and significance in some volume, function, and mass indices over time when they are exposed to normoxic conditions, which again highlights the Su/Hx models’ similarity to human PAH but an inability to fully recapitulate the disease. To investigate if Macitentan has any direct action on the RV, future CMR studies using specific models of RV failure like the pulmonary artery banding model (PAB), where there is no established lung disease that will confound results of any specific RV targeted treatment, will need to be utilized. As the PAB model involves placing a physical restriction to reduce the diameter of the artery (titanium clip) this may also remove the risk of the animals improving on their own.

## AUTHOR CONTRIBUTIONS

Gerard Murphy designed and performed experiments, analyzed, and interpreted data, and wrote the manuscript. Geeshath Jayasekera analyzed and interpreted MRI data. David J. Welsh designed experiments and interpreted data and wrote the manuscript. James Mullin and Lindsay Gallagher carried out MRI experiments. All authors were involved in critically revising the manuscript.

## CONFLICT OF INTEREST

The authors declare no conflict of interest.

## ETHICS STATEMENT

All experimental procedures were carried out in accordance with the United Kingdom Animal Procedures Act (1986), conforming to the guidelines from directive 2010/63/EU of the European Parliament on the protection of animals used for scientific purposes and with the “Guide for the Care and Use of Laboratory Animals” published by the US National Institutes of Health (NIH publication No. 85‐23, revised 1996).

## Supporting information

Supporting information.

Supporting information.

Supporting information.

Supporting information.
