# Supplementary material for: Exploring the failing right ventricle in pulmonary hypertension by cardiac magnetic resonance: An in vivo study utilizing Macitentan
Source: Pulm Circ. 2022 Jul 1;12(3):e12124. doi: 10.1002/pul2.12124 (PMC9438403; doi:10.1002/pul2.12124)
Supplement: Supplementary file 4 — Supporting information. [file PUL2-12-e12124-s001.docx]

**SUPPLEMENTARY MATERIAL**

**Exploring the failing right ventricle in pulmonary hypertension by CMR: An in vivo study utilising Macitentan.**

Gerard Murphy PhD^1^, Geeshath Jayasekera MBCHB^1^, James Mullin HNC^2^, Lindsay Gallagher HNC ^2^, David J Welsh PhD^1^

^1.^ Scottish Pulmonary Vascular Unit, Glasgow Caledonian University, Glasgow, G4 0BA, U.K. ^2.^ Institute of Neuroscience & Psychology, University of Glasgow, Glasgow, G12 8QQ, U.K.

**Supplementary Methods and Results**

***In vivo* study design**

All experimental procedures were carried out in accordance with the United Kingdom Animal Procedures Act (1986) and with the US NIH publication No. 85-23, revised 1996, and ethical approval was also granted by the University of Glasgow Ethics Committee. Rodents were housed in a 12-hour light dark cycle with access to food and water ad libitum. In vivo methodology is summarised in Figure S1. The Su/Hx group were administered a subcutaneous injection of Sugen 5416 [20mg/kg] suspended in vehicle of dH_2_O containing 0.5% carboxymethyl cellulose (w/v), 0.9% NaCl (w/v), 0.4% polysorbate 80 and0.9% benzyl alcohol (v/v). 4-week old Sprague Dawley rats (n=38) purchased from Envigo (Germany), were initially divided into two groups. A Sugen plus hypoxia group (Su/Hx) (n=24) and a control group (n=14). The Su/Hx group were administered a subcutaneous injection of Sugen 5416 [20mg/kg]. Rats were then placed for 3-weeks in hypobaric hypoxic conditions (550mbar), followed by two weeks of normoxic conditions (1013mbar) (Nx) to allow for PH development. Control (normoxic) group rats initially received a subcutaneous injection of the Sugen vehicle, then were kept in Nx conditions for 5 weeks. To demonstrate the development of PH, both control and Su/Hx rats (n=4 for each group) were assessed haemodynamically 5-weeks into the protocol. The remaining Su/Hx rats (n=20) subsequently received either Macitentan (30mg/kg) or vehicle (7.5% gelatin) via daily oral gavage for a further three weeks, control rats (n=10) only received vehicle. For CMR arm of the study, the rats were divided into Su/Hx with vehicle (n=6), Su/Hx with Macitentan (n=6) and control with vehicle (n=6). For the haemodynamic arm, the rats were divided into the same groups (n=4 per group). Figure S1 summarises the study design.

**Table S1: CMR variables between control, Su/Hx and Su/Hx + macitentan at baseline CMR scan.**

| **Baseline CMR** | **Control** | **Su/Hx** | **Su/Hx + Maci** |
| --- | --- | --- | --- |
| **RVEF (%)** | 62.34 ± 1.95 | 43.75 ± 2.75 | 37.29 ± 3.32 |
| **RVH** | 0.33 ± 0.02 | 0.58 ± 0.05 | 0.68 ± 0.04 |
| **RVESV (µL)** | 77.7 ± 5.51 | 150.79 ± 14.08 | 158.72 ± 10.20 |
| **RVESVi (µL/cm^2^)** | 0.17 ± 0.01 | 0.40 ± 0.04 | 0.43 ± 0.03 |
| **RVEDV (µL)** | 206.9 ± 12.24 | 266.7 ± 18.22 | 253.19 ± 11.08 |
| **RVEDVi (µL/cm^2^)** | 0.45 ± 0.03 | 0.70 ± 0.04 | 0.68 ± 0.03 |
| **RVSV (µL)** | 129.12 ± 9.08 | 115.91 ± 9.16 | 94.46 ± 8.99 |
| **RVSi (µL/cm^2^)** | 0.28 ± 0.02 | 0.30 ± 0.02 | 0.26 ± 0.02 |
| **RVCO (µL/min)** | 46378.9 ± 2915.8 | 42257.7 ± 3731.1 | 33702.23 ± 3177.4 |
| **RVCi (µL/min/cm^2^)** | 107.71 ± 7.74 | 111.05 ± 9.43 | 91.33 ± 8.79 |
| **RV Mass (mg)** | 119.42 ± 6.38 | 173.39 ± 15.95 | 191.62 ± 12.57 |
| **RV Mass Index (mg/cm^2^)** | 0.26 ± 0.01 | 0.46 ± 0.04 | 0.52 ± 0.04 |
| **LVEF (%)** | 53.94 ± 5.14 | 62.90 ± 2.48 | 52.03 ± 2.71 |
| **LVESV (µL)** | 165.95 ± 24.03 | 90.37 ± 5.64 | 102.43 ± 9.78 |
| **LVESVi (µl/cm^2^)** | 0.36 ± 0.05 | 0.24 ± 0.01 | 0.28 ± 0.03 |
| **LVEDV (µL)** | 355.2 ± 15.62 | 244.79 ± 8.16 | 212.32 ± 12.07 |
| **LVEDVi (µL/cm^2^)** | 0.78 ± 0.02 | 0.64 ± 0.01 | 0.57 ± 0.03 |
| **LVSV (µL)** | 189.21 ± 15.87 | 154.42 ± 9.32 | 109.88 ± 6.98 |
| **LVSVi (µL/cm^2^)** | 0.42 ± 0.03 | 0.41 ± 0.02 | 0.29 ± 0.02 |
| **LVCO (µL/min)** | 67952.8 ± 5412.9 | 56280.03 ± 4155.6 | 39146.33 ± 2197.0 |
| **LVCi (µL/min/cm^2^)** | 149.63 ± 11.68 | 147.74 ± 9.94 | 106.23 ± 7.48 |
| **LV Mass (mg)** | 363.5 ± 15.85 | 298.95 ± 11.49 | 284.75 ± 15.61 |
| **LV Mass index (mg/cm^2^)** | 0.79 ± 0.02 | 0.78 ± 0.02 | 0.77 ± 0.04 |

Table S1: Ventricular masses and volumes give as µL and mg and µL/cm2 and mg/cm2 when indexed to total body surface area (TBSA).

**Table S2: CMR variables between control + vehicle, Su/Hx + vehicle and Su/Hx + macitentan at 2^nd^ CMR scan.**

| **2-week CMR** | **Control + Veh** | **Su/Hx + Veh** | **Su/Hx + Maci** |
| --- | --- | --- | --- |
| **RVEF (%)** | 61.20 ± 3.7 | 47.99 ± 3.4 | 52.36 ± 2.5 |
| **RVH** | 0.33 ± 0.01 | 0.53 ± 0.04 | 0.56 ± 0.03 |
| **RVESV (µL)** | 86.34 ± 8.39 | 125.47 ± 14.60 | 110.07 ± 6.29 |
| **RVESVi (µL/cm^2^)** | 0.18 ± 0.01 | 0.31 ± 0.04 | 0.28 ± 0.02 |
| **RVEDV (µL)** | 223.77 ± 11.72 | 238.94 ± 17.77 | 232.52 ± 8.51 |
| **RVEDVi (µL/cm^2^)** | 0.48 ± 0.02 | 0.59 ± 0.04 | 0.58 ± 0.02 |
| **RVSV (µL)** | 137.43 ± 11.57 | 113.46 ± 10.17 | 121.91 ± 7.42 |
| **RVSi (µL/cm^2^)** | 0.29 ± 0.02 | 0.28 ± 0.02 | 0.31 ± 0.02 |
| **RVCO (µL/min)** | 48827.69 ± 4362.5 | 39157.68 ± 4430.6 | 44830 ± 3326.7 |
| **RVCi (µL/min/cm^2^)** | 104.73 ± 9.47 | 96.82 ± 9.16 | 112.22 ± 8.02 |
| **RV Mass (mg)** | 123.36 ± 4.94 | 170.74 ± 14.27 | 170.8 ± 9.65 |
| **RV Mass Index (mg/cm^2^)** | 0.26 ± 0.01 | 0.43 ± 0.04 | 0.43 ± 0.03 |
| **LVEF (%)** | 53.81 ± 3.71 | 57.24 ± 1.87 | 54.35 ± 1.99 |
| **LVESV (µL)** | 168.19 ± 21.07 | 105.07 ± 4.6 | 118.68 ± 5.65 |
| **LVESVi (µl/cm^2^)** | 0.36 ± 0.04 | 0.26 ± 0.01 | 0.30 ± 0.01 |
| **LVEDV (µL)** | 359.17 ± 24.19 | 246.84 ± 11.29 | 260.85 ± 10.06 |
| **LVEDVi (µL/cm^2^)** | 0.76 ± 0.04 | 0.61 ± 0.02 | 0.65 ± 0.02 |
| **LVSV (µL)** | 190.97 ± 14.36 | 141.77 ± 9.59 | 142.17 ± 8.99 |
| **LVSVi (µL/cm^2^)** | 0.41 ± 0.02 | 0.35 ± 0.02 | 0.36 ± 0.02 |
| **LVCO (µL/min)** | 67655.2 ± 4560.7 | 48950.24 ± 4591.8 | 51923.5 ± 2746.3 |
| **LVCi (µL/min/cm^2^)** | 144.46 ± 7.09 | 121.26 ± 9.49 | 130.26 ± 7.29 |
| **LV Mass (mg)** | 376.79 ± 21.72 | 322.63 ± 12.95 | 304.75 ± 12.54 |
| **LV Mass index (mg/cm^2^)** | 0.80 ± 0.02 | 0.80 ± 0.02 | 0.76 ± 0.03 |

Table S2: Ventricular masses and volumes give as µL and mg and µL/cm^2^ and mg/cm^2^ when indexed to total body surface area (TBSA).

**Table S3: CMR variables between control + vehicle, Su/Hx + vehicle and Su/Hx + Macitentan at 3^rd^ CMR scan.**

| **3-week CMR** | **Control + Veh** | **Su/Hx + Veh** | **Su/Hx + Maci** |
| --- | --- | --- | --- |
| **RVEF (%)** | 62.21 ± 3.50 | 47.98 ± 3.26 | 51.15 ± 2.36 |
| **RVH** | 0.34 ± 0.02 | 0.52 ± 0.04 | 0.51 ± 0.04 |
| **RVESV (µL)** | 74.82 ± 6.55 | 124.56 ± 15.65 | 107.09 ± 8.51 |
| **RVESVi (µL/cm^2^)** | 0.16 ± 0.01 | 0.31 ± 0.04 | 0.27 ± 0.02 |
| **RVEDV (µL)** | 198.71 ± 7.72 | 235.36 ± 17.85 | 218.62 ± 12 |
| **RVEDVi (µL/cm^2^)** | 0.42 ± 0.01 | 0.58 ± 0.05 | 0.54 ± 0.03 |
| **RVSV (µL)** | 123.87 ± 8.56 | 110.80 ± 6.41 | 111.53 ± 7.27 |
| **RVSi (µL/cm^2^)** | 0.26 ± 0.02 | 0.27 ± 0.01 | 0.28 ± 0.01 |
| **RVCO (µL/min)** | 45829.08 ± 3248.6 | 40286.88 ± 1936.9 | 39729.79 ± 3197.9 |
| **RVCi (µL/min/cm^2^)** | 97.85 ± 7.62 | 98.48 ± 3.80 | 98.53 ± 6.59 |
| **RV Mass (mg)** | 123.05 ± 3.76 | 168.96 ± 13.50 | 154.84 ± 9.35 |
| **RV Mass Index (mg/cm^2^)** | 0.26 ± 0.01 | 0.41 ± 0.04 | 0.39 ± 0.02 |
| **LVEF (%)** | 50.59 ± 3.39 | 58.45 ± 1.82 | 54.91 ± 2.60 |
| **LVESV (µL)** | 166.16 ± 20.79 | 113.46 ± 6.67 | 114.39 ± 4.82 |
| **LVESVi (µl/cm^2^)** | 0.35 ± 0.04 | 0.28 ± 0.02 | 0.28 ± 0.01 |
| **LVEDV (µL)** | 330.79 ± 21.75 | 272.96 ± 10.58 | 256.02 ± 13.46 |
| **LVEDVi (µL/cm^2^)** | 0.70 ± 0.03 | 0.66 ± 0.02 | 0.63 ± 0.03 |
| **LVSV (µL)** | 164.62 ± 8.99 | 159.50 ± 7.77 | 141.62 ± 12.51 |
| **LVSVi (µL/cm^2^)** | 0.35 ± 0.02 | 0.39 ± 0.01 | 0.35 ± 0.03 |
| **LVCO (µL/min)** | 60876.97 ± 3242.6 | 58280.63 ± 3254.38 | 50534.12 ± 5184.4 |
| **LVCi (µL/min/cm^2^)** | 129.43 ± 6.12 | 142.10 ± 4.96 | 125.48 ± 12.14 |
| **LV Mass (mg)** | 368.66 ± 26.63 | 326.50 ± 14.07 | 302.62 ± 15.01 |
| **LV Mass index (mg/cm^2^)** | 0.78 ± 0.04 | 0.79 ± 0.03 | 0.75 ± 0.04 |

Table S3: Ventricular masses and volumes give as µL and mg and µL/cm2 and mg/cm2 when indexed to total body surface area (TBSA).

**Table S4: Inter-observer variability of CMR analysis.**

| **Measurement** | **Intraclass correlation coefficient** | **95% Confidence interval** |
| --- | --- | --- |
| **RVEF** | 0.995 | 0.969-0.998 |
| **RVH** | 0.992 | 0.954-0.998 |
| **RVESV** | 0.995 | 0.934-0.998 |
| **RVEDV** | 0.984 | 0.768-0.998 |
| **RVSV** | 0.985 | 0.921-0.997 |
| **RVCO** | 0.986 | 0.928-0.998 |
| **RV Mass** | 0.991 | 0.952-0.998 |
| **LVEF** | 0.964 | 0.573-0.993 |
| **LVESV** | 0.997 | 0.757-0.999 |
| **LVEDV** | 0.997 | 0.984-0.998 |
| **LVSV** | 0.989 | 0.936-0.998 |
| **LVCO** | 0.987 | 0.928-0.998 |
| **LV Mass** | 0.990 | 0.927-0.998 |

Table S4: Assessed by intra-class correlation coefficient (ICC) using a two-way mixed effects model with absolute agreement and average measures.

**Figure S1: Outline of Su/Hx in vivo study with CMR and haemodynamics arms**


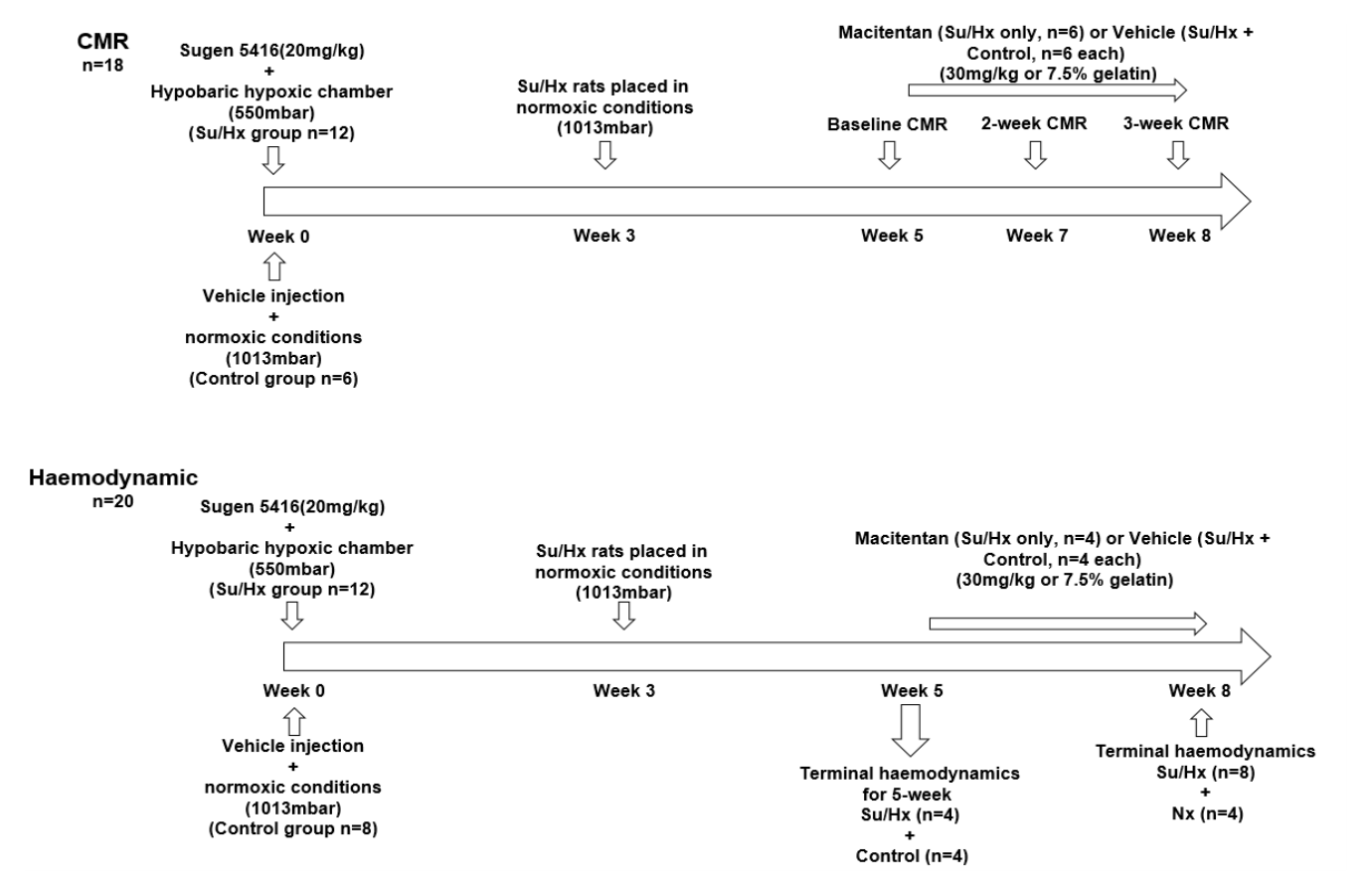


At week 0 Sprague-Dawley rats received either subcutaneous injection of sugen 5416 (20mg/kg) before being placed in hypobaric chamber (Su/Hx) or an injection of sugen vehicle then placed under normoxic conditions (Control). At week 3, Su/Hx rats are removed from the hypobaric chamber and placed under normoxic conditions. At week 5 in the CMR group, control and Su/Hx rats underwent a baseline CMR after which daily treatment by oral gavage of either Macitentan (30mg/kg) or vehicle was commenced. Further CMR scans were carried out at two and three weeks of treatment. Separately, at week 5 in the haemodynamic group, control and Su/Hx rats underwent terminal haemodynamic measurement to check the establishment of PH. The remaining control and Su/Hx rats were dosed with either Macitentan (30mg/kg) or vehicle for three weeks before terminal haemodynamics were determined.

**Figure S2: Representative CMR cine images of the heart of a control rat.**


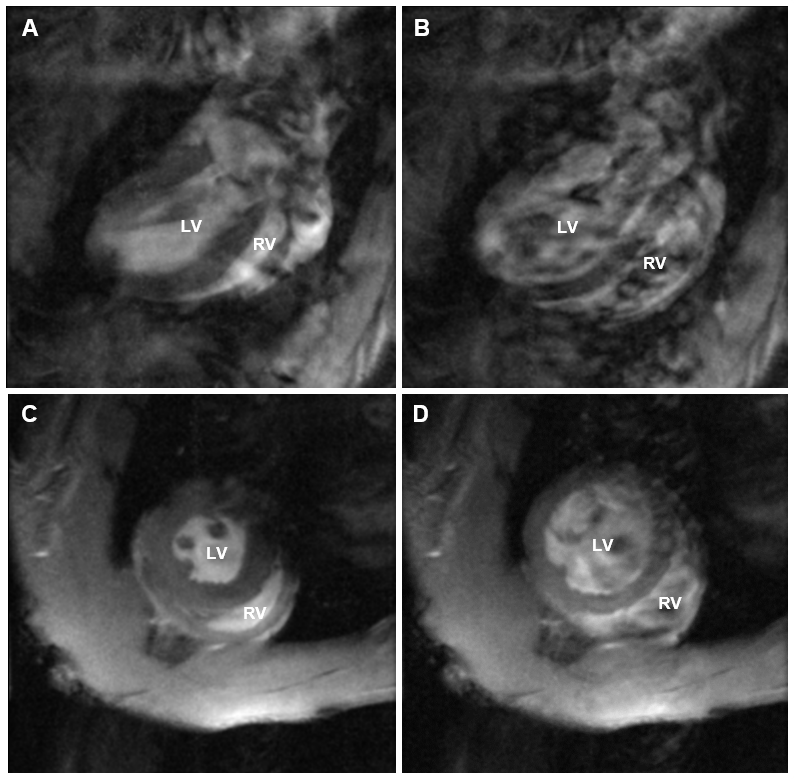


(A, B) long axis at systole and diastole and (C, D) short axis at both systole and diastole from a control rat, identifying both left ventricle (LV) and right ventricle (RV). Long axis cine scans were carried in order to set the angle of the short axis cine scan. Short axis cine images were acquired using a slice thickness of 1.5mm ensuring the entirety of biventricular length was covered.

**Figure S3: Inter-group comparison using CMR determining the effect of Su/Hx on RV ejection fraction and RV hypertrophy (RV/LV+S) at baseline CMR.**


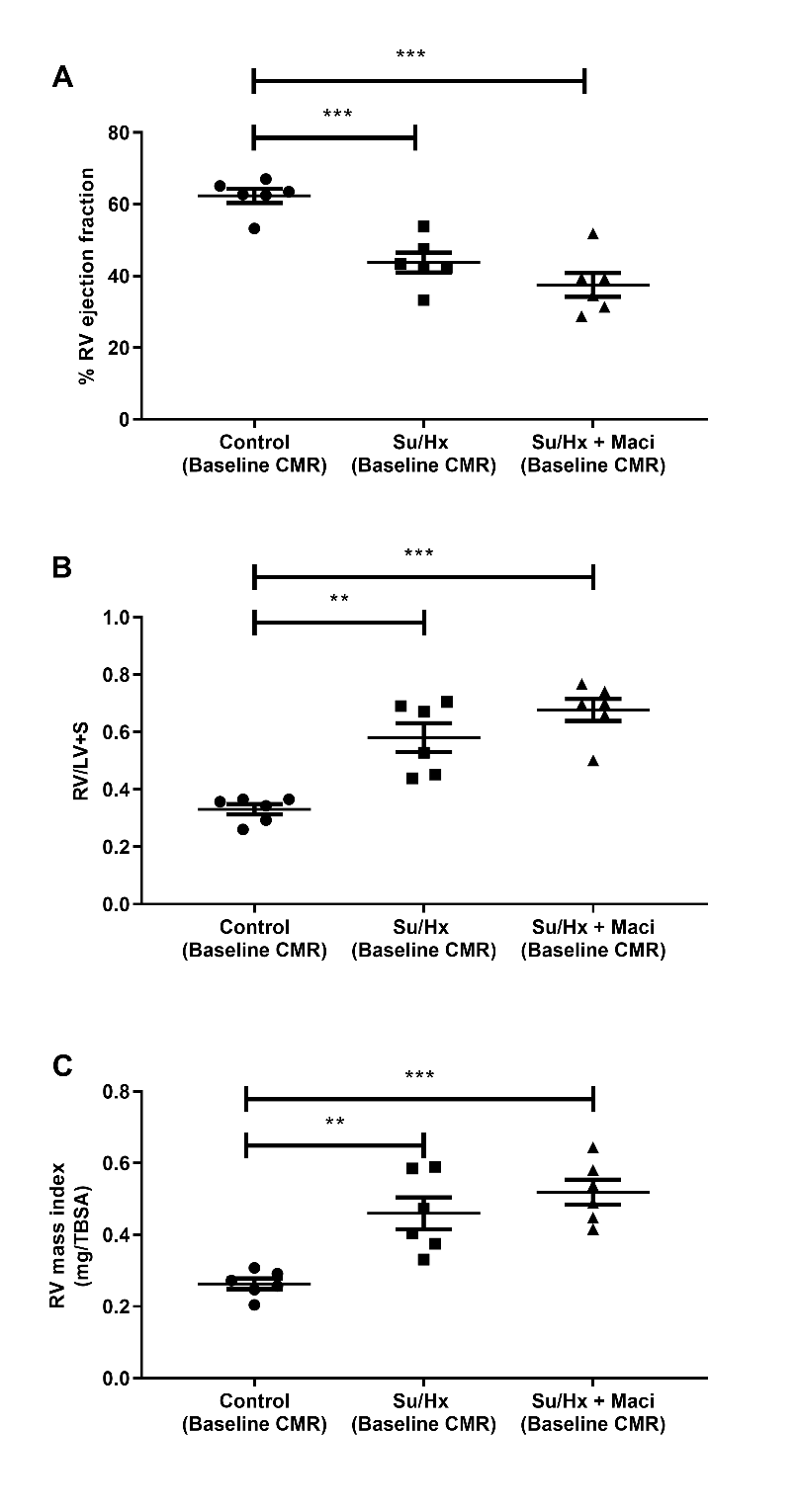


(A) RV ejection fraction and (B) RV hypertrophy (C) RV mass index. Data represented as mean ± SEM. n=6, **p<0.01, ***p<0.001, as indicated, determined by one-way analysis of variance with Tukey’s post hoc analysis.

**Figure S4: Comparison of control and Su/Hx rats after 5-weeks measured by terminal haemodynamics.**


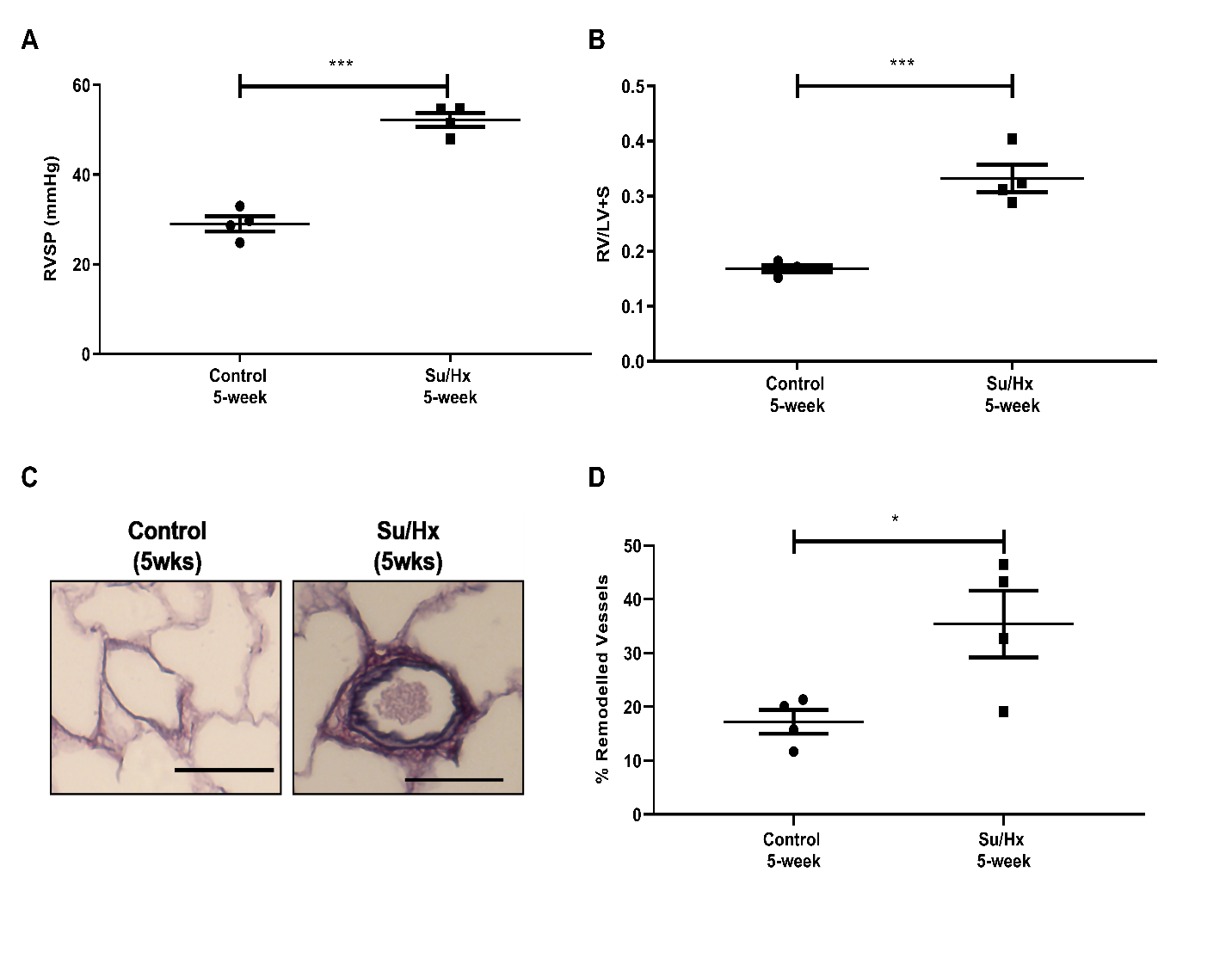


(A) RVSP, (B) RV hypertrophy (RV/LV+S), (C) representative micrograph of control 5-week, Su/Hx 5-week, (D) % remodelling. Data represented as mean ± SEM. n=4, *p<0.05, ***p<0.001, as indicated, determined by 2‐tailed Student's unpaired t‐test. Scale bar = 50µm.

**Figure S5: Inter-group comparison of control and Su/Hx rats after 8-week protocol measured by terminal haemodynamic.**


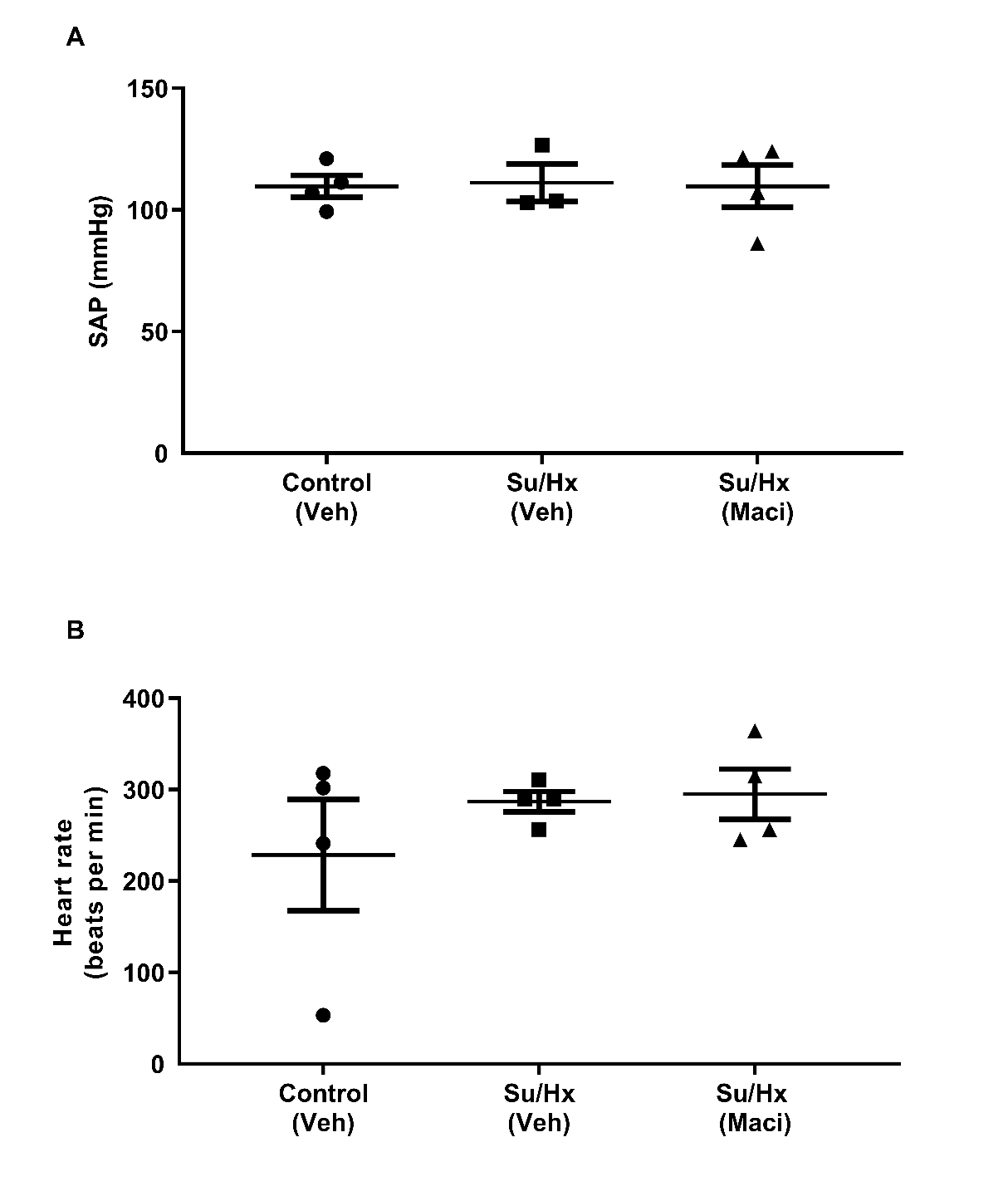


(A) systemic arterial pressure (SAP) and (B) heart rate. Data represented as mean ± SEM. n=3-4, determined by one-way analysis of variance with Tukey’s post hoc analysis.

**Figure S6: Inter-group comparison using CMR determining the effect of Su/Hx on RV ejection fraction and RV hypertrophy (RV/LV+S) at 3-week CMR.**

**
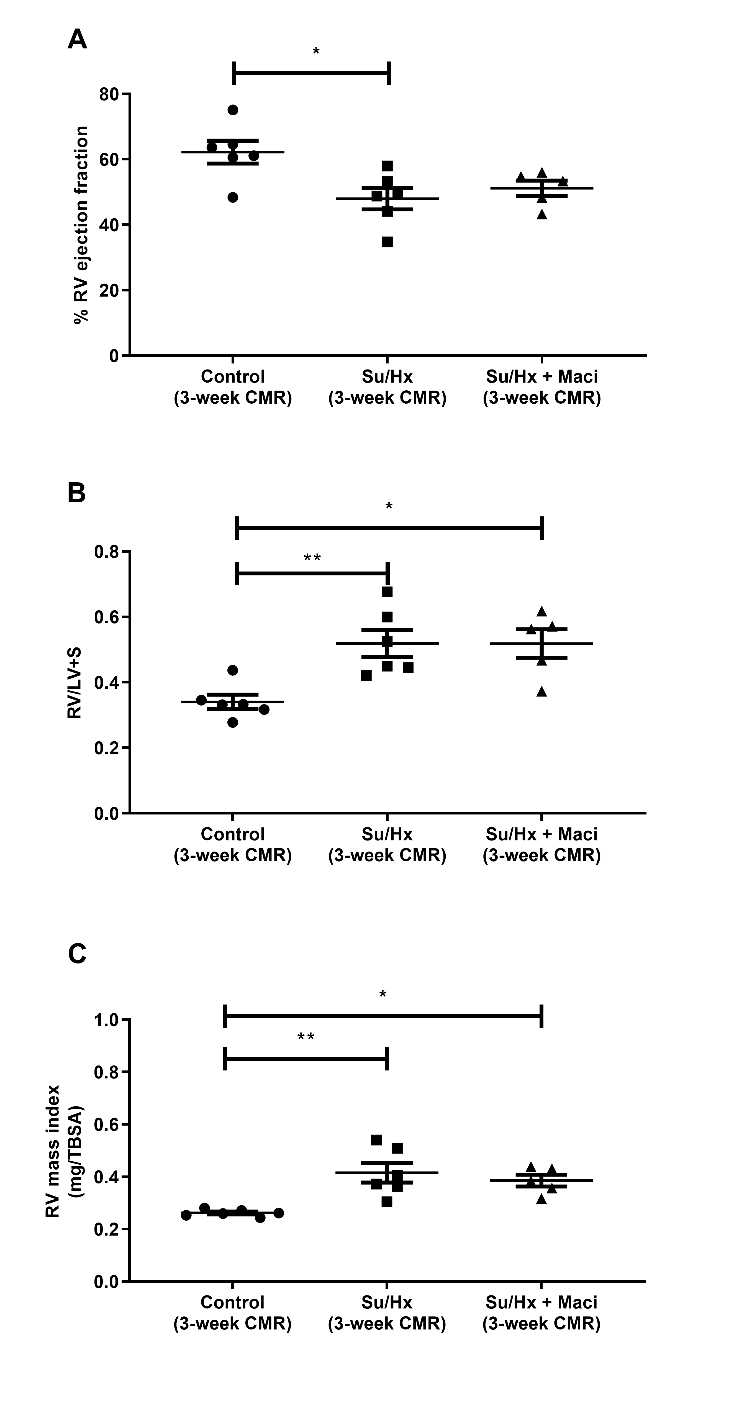
**

(A) RV ejection fraction and (B) RV hypertrophy (C) RV mass index. Data represented as mean ± SEM. n=5-6, *p<0.05, **p<0.01, as indicated, determined by one-way analysis of variance with Tukey’s post hoc analysis.

**Figure S7: Repeat CMR analysis of the effect of Macitentan (Maci) or vehicle (Veh) on right ventricular stroke volume index (RVSi) and cardiac index (RVCi).**


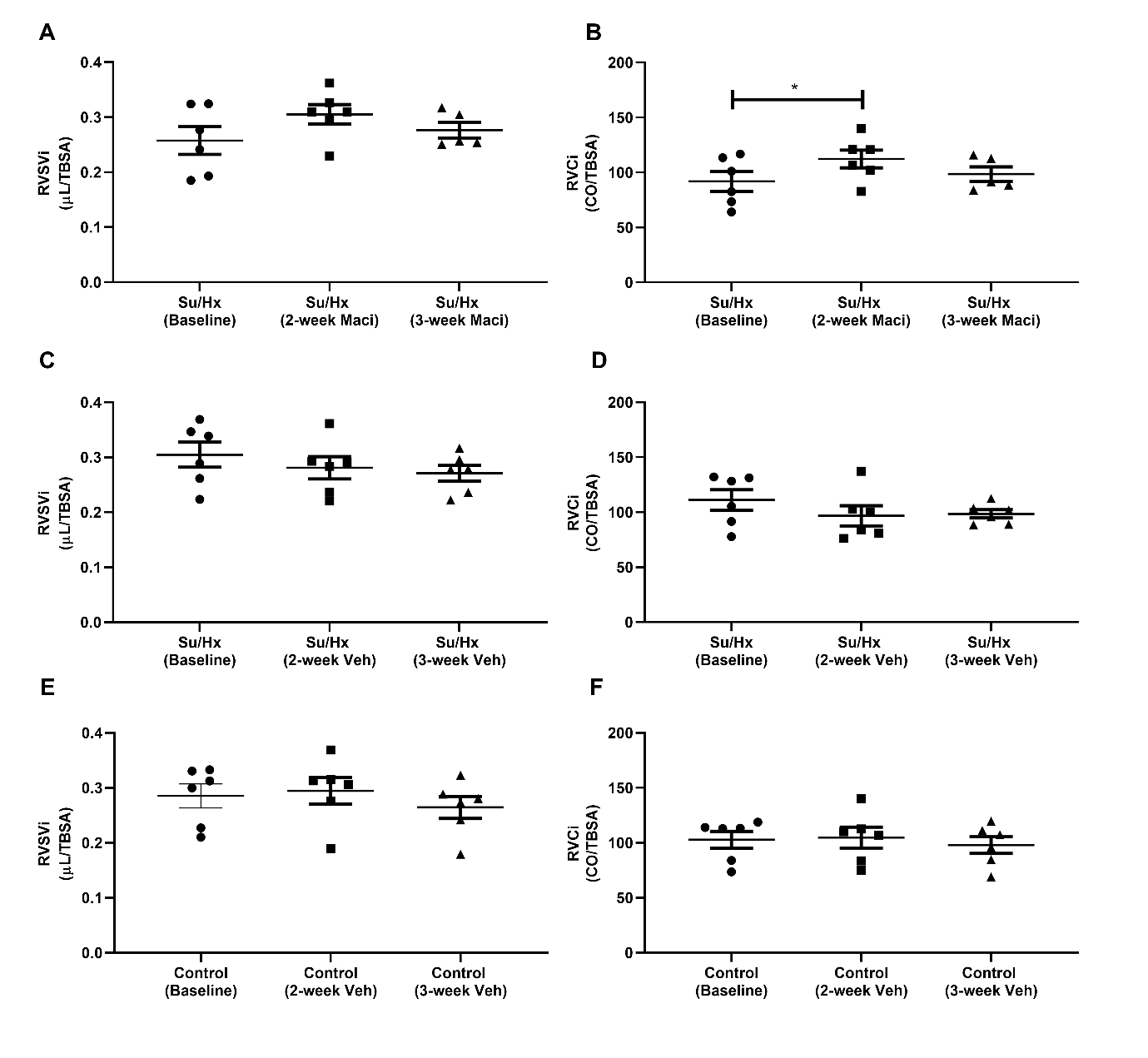


Volumes are indexed to total body surface area (TBSA) (cm^2^). Su/Hx with Macitentan (A) RVSi and (B) RVCi. Su/Hx vehicle (C) RVSi and (D) RVCi. Control vehicle (E) RVSi and (F) RVCi. Data represented as mean ± SEM. n=5-6, *p<0.05, determined by repeated measures one-way analysis of variance or mixed effects model with Tukey’s post hoc analysis.

**Figure S8: Repeat CMR analysis of the effect of macitentan (Maci) or vehicle (Veh) on LV ejection fraction.**


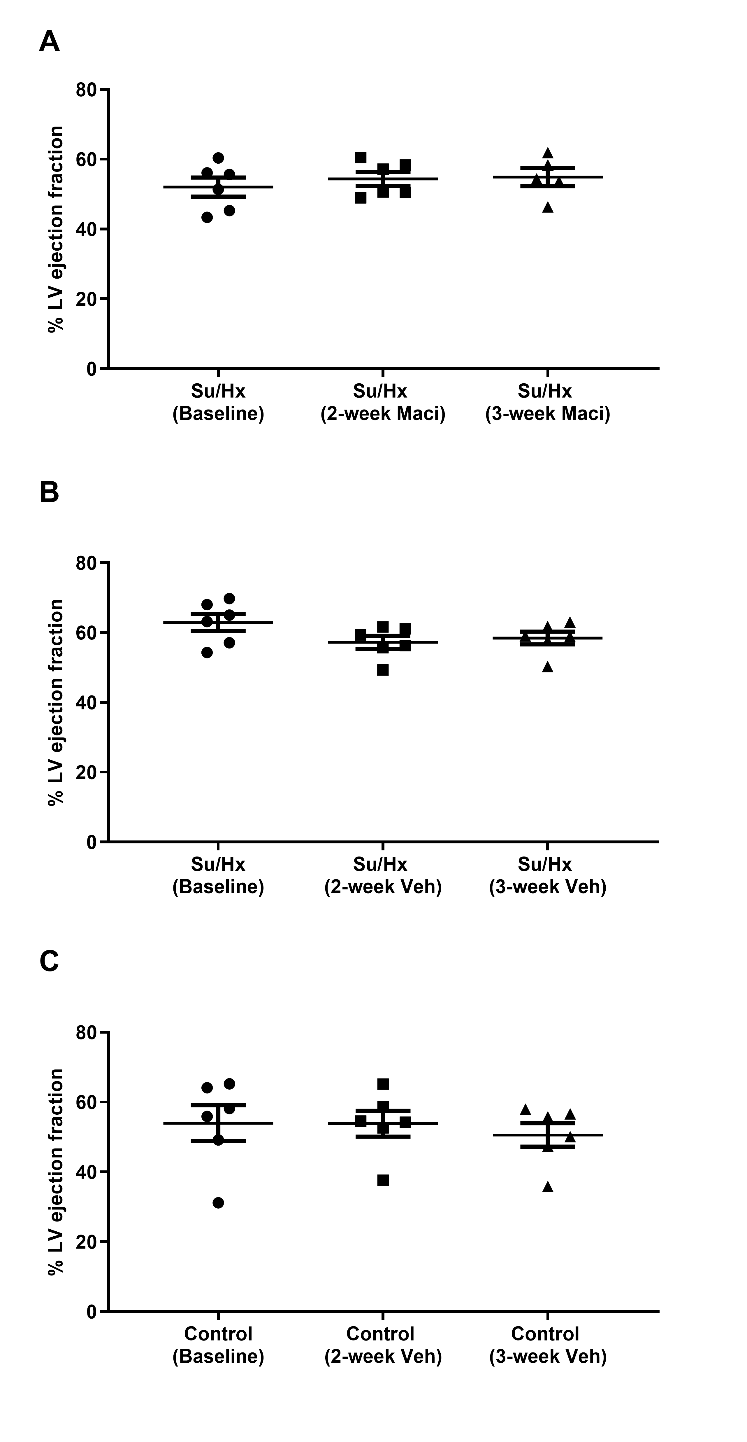


Su/Hx with Macitentan (A) LV ejection fraction. Su/Hx vehicle (B) LV ejection fraction. Control vehicle (C) LV ejection fraction. Data represented as mean ± SEM. n=5-6, as indicated, determined by repeated measures one-way analysis of variance or mixed effects model with Tukey’s post hoc analysis.

**Figure S9: Repeat CMR analysis of the effect of macitentan or vehicle on left ventricular end systolic volume index (LVESVi) and end diastolic volume (LVEDVi).**


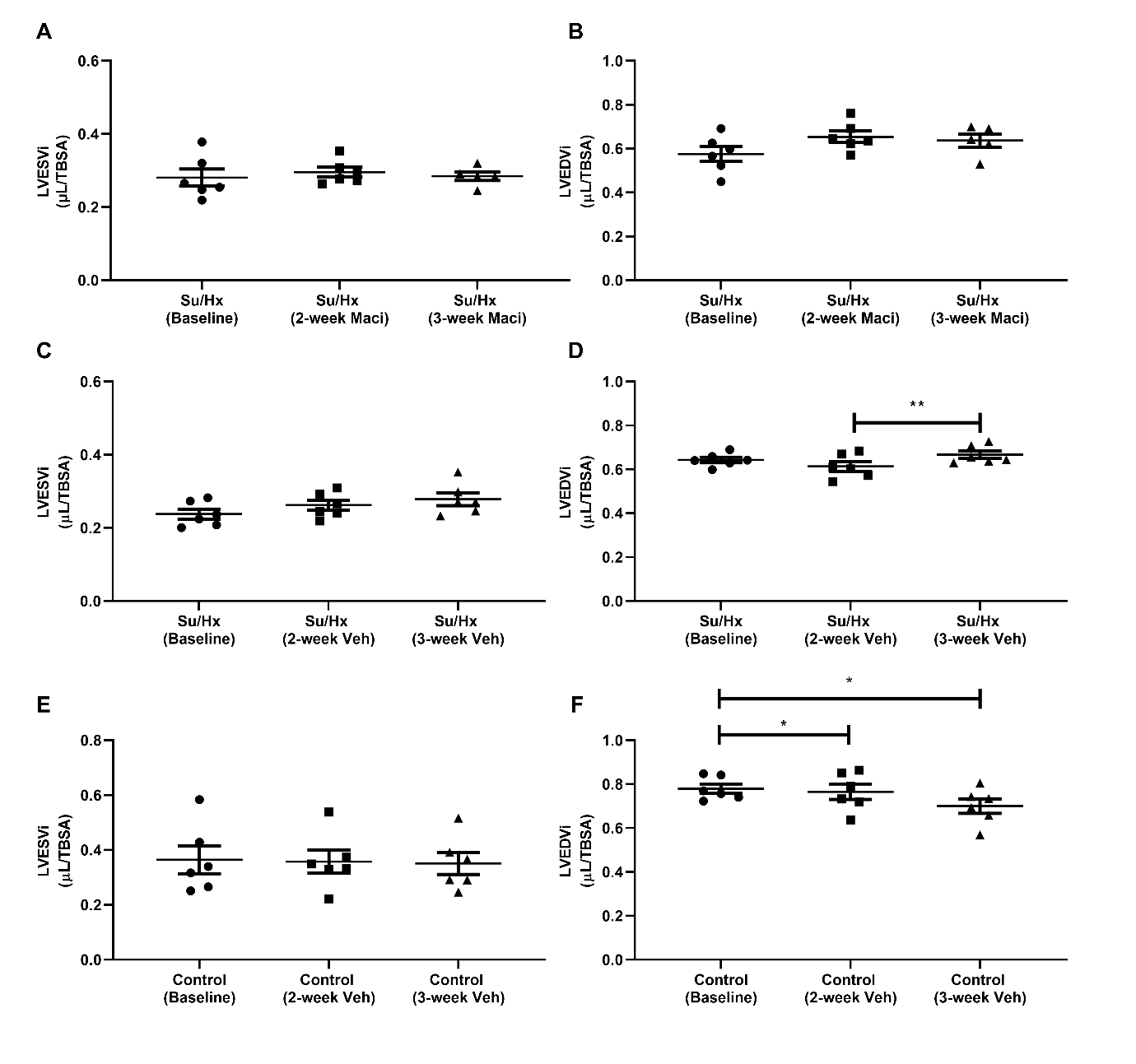


Volumes are indexed to total body surface area (TBSA) (cm^2^). Su/Hx with macitentan (A) LVESVi and (B) LVEDVi. Su/Hx vehicle (C) LVESVi and (D) LVEDVi. Control vehicle (E) LVESVi and (F) LVEDVi. Data represented as mean ± SEM. n=5-6, *p<0.05, **p<0.01 as indicated, determined by repeated measures one-way analysis of variance or mixed effects model with Tukey’s post hoc analysis.

**Figure S10: Repeat CMR analysis of the effect of macitentan (Maci) or vehicle (Veh) on left ventricular stroke volume index (LVSVi) and cardiac index (LVCi).**


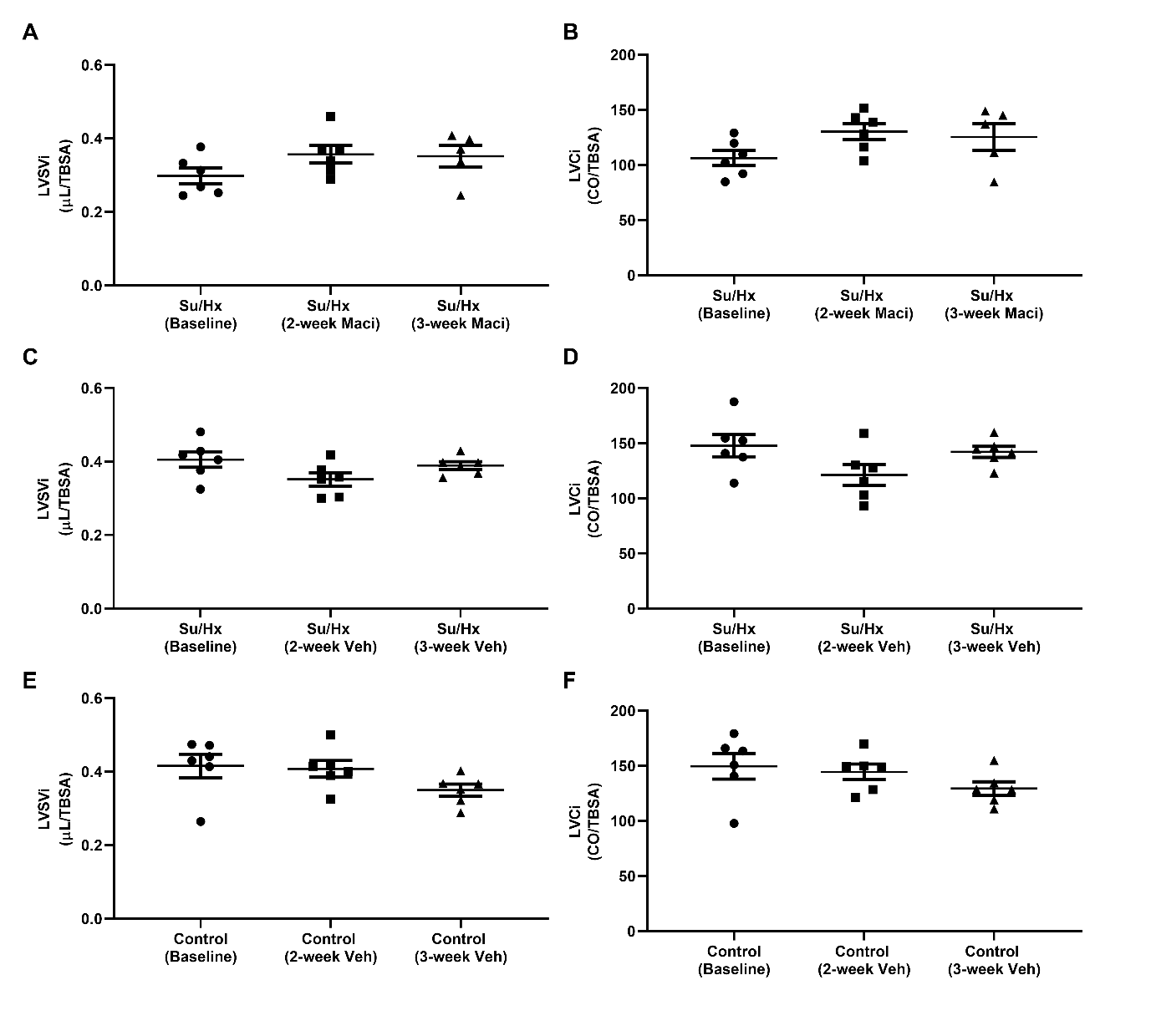


Volumes are indexed to total body surface area (TBSA) (cm2). Su/Hx with macitentan (A) LVSi and (B) LVCi. Su/Hx vehicle (C) LVSi and (D) LVCi. Control vehicle (E) LVSi and (F) LVCi. Data represented as mean ± SEM. n=5-6, *p<0.05 determined by repeated measures one-way analysis of variance or mixed effects model with Tukey’s post hoc analysis.

**Figure S11: Inter-group comparison using CMR analysis of the effect of macitentan on left ventricular eccentricity index (LVEI).**


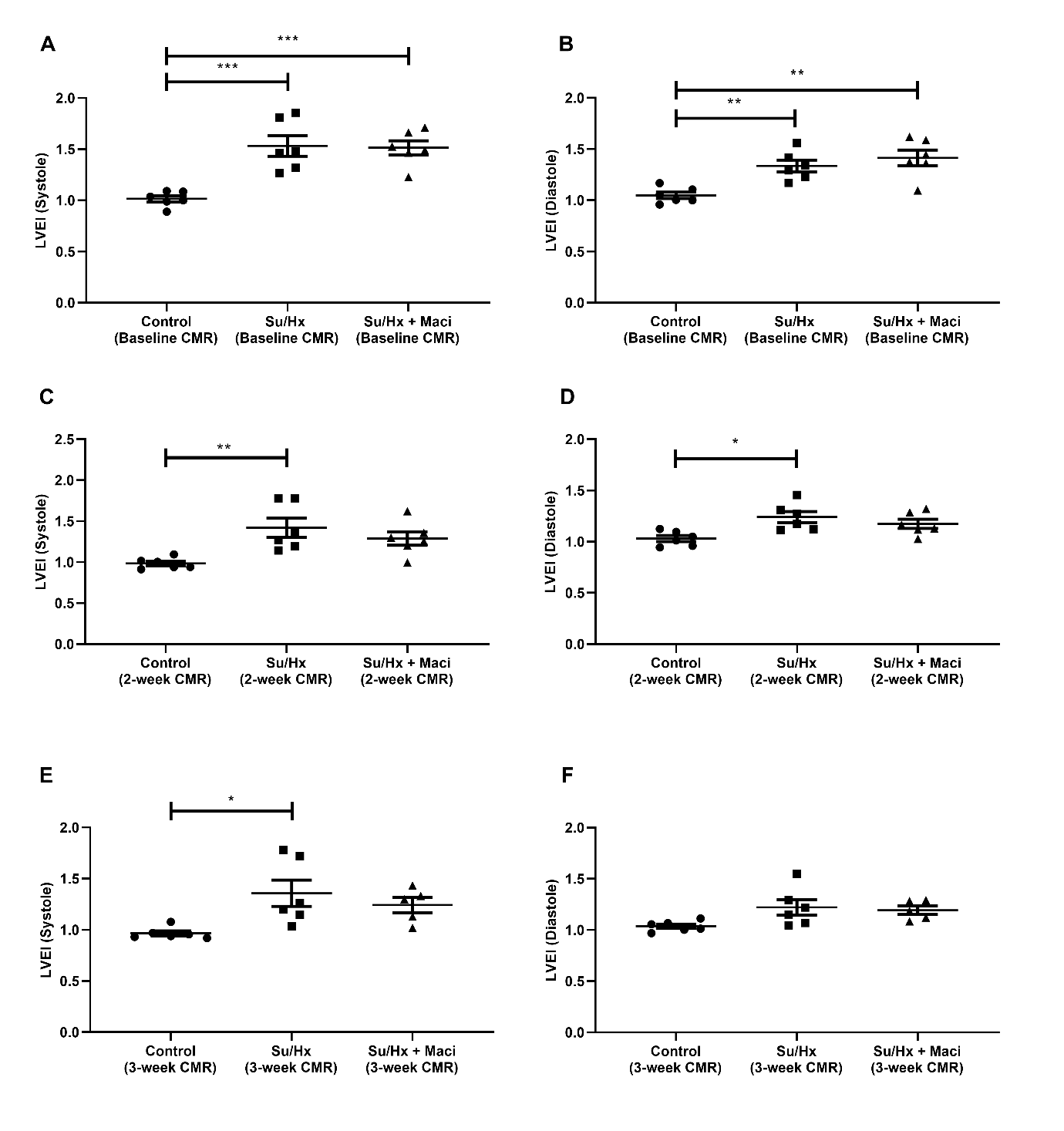


(A) LVEI Systole and (B) diastole at baseline CMR, (C) LVEI Systole and (D) diastole at 2-week CMR, (E) LVEI Systole and (F) diastole at 3-week CMR. Data represented as mean ± SEM. n=5-6, *p<0.05, **p<0.01, ***p<0.001 as indicated, determined by one-way analysis of variance model with Tukey’s post hoc analysis.

**Figure S13: Analysis of body weight and total body surface area (TBSA) (cm^2^) in rats undergoing repeat CMR.**


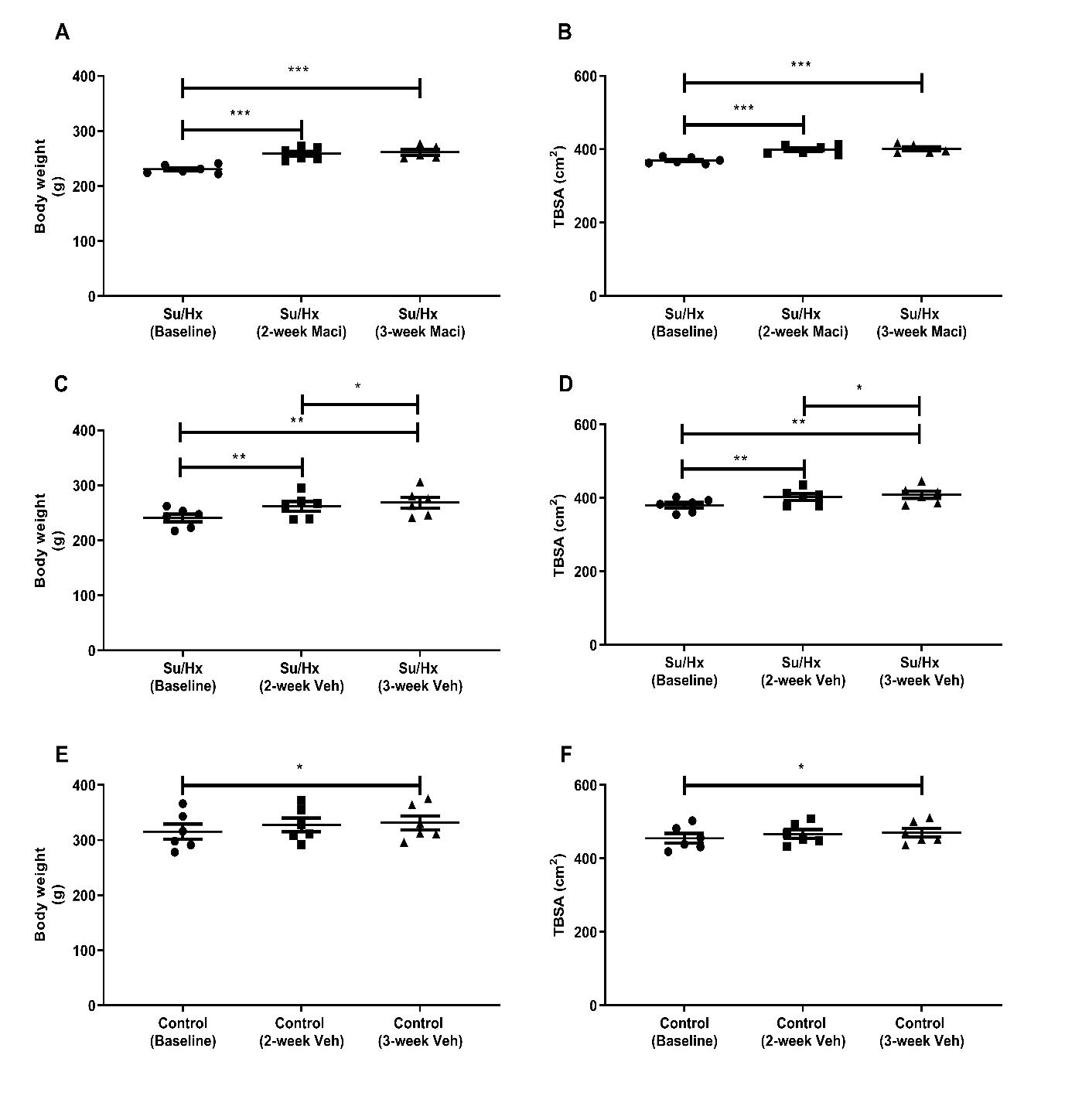
 Su/Hx with macitentan (Maci) (A) body weight and (B) TBSA. Su/Hx vehicle (C) body weight and (D) TBSA. Control vehicle (Veh) (E) body weight and (F) TBSA. Data represented as mean ± SEM. n=5-6, determined by repeated measures one-way analysis of variance or mixed effects model with Tukey’s post hoc analysis.

**Video S1: Representative cine video of short axis repeat CMRs of a control rat**

Video S1: (A) baseline, (B) 2-week and (C) 3-week CMRs of a control rat. Left ventricle (LV) and right ventricle (RV) identified in baseline scan. Short axis cine videos were acquired using a slice thickness of 1.5mm ensuring the entirety of biventricular length was covered.

**Video S2: Representative cine video of short axis repeat CMRs of a Su/Hx rat**

Video S2: (A) baseline, (B) 2-week and (C) 3-week CMRs of a Su/Hx rat treated with vehicle. Left ventricle (LV) and right ventricle (RV) identified in baseline scan. Short axis cine videos were acquired using a slice thickness of 1.5mm ensuring the entirety of biventricular length was covered.

**Video S3: Representative cine video of short axis repeat CMRs of a Su/Hx rat**

Video S3: (A) baseline, (B) 2-week and (C) 3-week CMRs of a Su/Hx treated with Macitentan rat. Left ventricle (LV) and right ventricle (RV) identified in baseline scan. Short axis cine videos were acquired using a slice thickness of 1.5mm ensuring the entirety of biventricular length was covered.
